# A Method for Quantifying, Visualising, and Analysing Gastropod Shell Form

**DOI:** 10.1371/journal.pone.0157069

**Published:** 2016-06-09

**Authors:** Thor-Seng Liew, Menno Schilthuizen

**Affiliations:** 1 Institute for Tropical Biology and Conservation, Universiti Malaysia Sabah, Jalan UMS, 88400, Kota Kinabalu, Sabah, Malaysia; 2 Institute Biology Leiden, Leiden University, P.O. Box 9516, 2300 RA, Leiden, The Netherlands; 3 Naturalis Biodiversity Center, P.O. Box 9517, 2300 RA, Leiden, The Netherlands; University of California, UNITED STATES

## Abstract

Quantitative analysis of organismal form is an important component for almost every branch of biology. Although generally considered an easily-measurable structure, the quantification of gastropod shell form is still a challenge because many shells lack homologous structures and have a spiral form that is difficult to capture with linear measurements. In view of this, we adopt the idea of theoretical modelling of shell form, in which the shell form is the product of aperture ontogeny profiles in terms of aperture growth trajectory that is quantified as curvature and torsion, and of aperture form that is represented by size and shape. We develop a workflow for the analysis of shell forms based on the aperture ontogeny profile, starting from the procedure of data preparation (retopologising the shell model), via data acquisition (calculation of aperture growth trajectory, aperture form and ontogeny axis), and data presentation (qualitative comparison between shell forms) and ending with data analysis (quantitative comparison between shell forms). We evaluate our methods on representative shells of the genera *Opisthostoma* and *Plectostoma*, which exhibit great variability in shell form. The outcome suggests that our method is a robust, reproducible, and versatile approach for the analysis of shell form. Finally, we propose several potential applications of our methods in functional morphology, theoretical modelling, taxonomy, and evolutionary biology.

## Introduction

### Empirical and theoretical approaches in the study of shell form

The external form diversity of organisms is the most obvious evidence for their evolution, and thus is a key element in most branches of biology. The molluscan shell has been a popular example in morphological evolution studies because it is geometrically simple, yet diverse in form. The shell form is controlled by the shell ontogenetic process, which follows a simple accretionary growth mode where new shell material is accumulatively deposited to the existing aperture. The evolution of shell forms has been studied either by using empirical approaches that focus on the quantification of actual shell forms or by using theoretical approaches that focus on the simulation of shell ontogenetic processes and geometric forms.

Notwithstanding the active development in both empirical and theoretical approaches to the study of shell form, there has been very little integration between both schools. For the empirical approach, the quantification methods of shell form have evolved from traditional linear measurement to landmark-based geometric morphometrics and outline analyses (for an overview see [1]). At the same time, for the theoretical approach, the simulations of shell form have evolved from simple geometry models that aimed to reproduce the form, to more comprehensive models that simulate shell ontogenetic processes (for an overview see [2]). Hence, each of the two approaches has been moving forward but away from each other, where synthesis between the two schools of shell morphologists has become more challenging.

In empirical morphological studies, shell form, either in terms of heights and widths in traditional morphometrics or in terms of geometry of procrustes distances in geometric morphometrics, is quantified by a set of homologous reference points or landmarks on the shell, which can be easily obtained from the fixed dimensions of the shell. Thus, both methods could abstract the shell form in terms of size and shape of the particular shell dimensions, and the between-sample variation of shell size and shape can be assessed (in most cases only within one study). On the other hand, it is not possible to reconstruct the actual shell form from these quantitative measurements, because the shell’s accretionary growth model and spiral geometry cannot be quantified on the basis of arbitrary reference points or fixed dimensions [3]. Nevertheless, the traditional and geometric morphometric methods have been accepted widely as standard quantification methods for shell form in many different fields of research.

In contrast to empirical morphometrics in which the aim is to quantify the actual shell, theoretical morphologists focus on the simulation of an accretionary growth process which produces a shell form that is similar to actual shells. This field was established with the theoretical shell model of D.M. Raup [4,5]. Within the first two decades after these publications, only a few different versions of shell models were proposed [6–10]. The subsequent two decades, thanks to the popularity and power of desktop computing, many more theoretical shell models were published [11–25]. Finally, we saw further improvements in the published theoretical models in recent years. These recent models simulate shell forms that more accurately resemble actual shells because of improved programming software, more complex algorithms, and advancement of 3D technology [2,26–33]. Here, we will not further discuss the details of the at least 29 published shell models, but refer to the comprehensive overviews and descriptions of these models in Urdy et al. [2] and Dera et al. [34].

In brief, the latest theoretical shell models are able to simulate irregularly-coiled shell forms and ornamentations that resemble actual shells, whereas the earlier models could only simulate the regular and general shape of shells. The major refinements that have been made during the almost five decades’ development of theoretical shell models are the following modifications of the algorithm: (1) from a fixed reference frame to a moving reference frame system; (2) from modelling based on numerical geometry parameters to growth-parameter-based modelling (e.g. growth rates); (3) from three parameters to more than three parameters, which has made fine-tuning of the shell simulation (e.g. aperture shape) possible. The key element of the theoretical modelling of shells is the generation of shell form by simulating the aperture ontogeny in terms of growth trajectory and form along the shell ontogeny. Hence, this has an advantage over the empirical approach in the numerical representation of the shell geometry form in terms of the 3D quantification and the actual shell ontogenetic processes [35].

Since the empirical and theoretical researchers studying shell form with two totally different quantification methods, our understanding of shell evolution cannot progress solely by using either empirical morphometrics or theoretical models. Ideally, theoretical models need to be evaluated by empirical data of shell morphometrics, and, vice-versa, empirical morphometric methods need to be improved to obtain data that better reflect the actual shell form and morphogenesis which can then be used to improve the theoretical models (see also [36]). In this dilemma lies the central problem of shell form quantification and it urgently needs to be addressed in order to integrate and generalise studies of shell form evolution.

### Empirical studies rarely use theoretical shell models

Despite the fact that, since the 1980s, many shell models have been published that are more complex and versatile, the first theoretical shell model of Raup still remains the most popular. There were many attempts by empirical morphologists to use the original or a modified version of Raup’s parameters to quantify natural shell forms [37–53]. Surprisingly, all the other shell models, many of which produce more realistic forms, have received very little attention as compared to Raup’s model (see [35, 54–56] for exceptions). This ironic situation might be explained by the elegance and generality of Raup’s model that is intuitively and mathematically simple to be used by empirical morphologists (mostly biologists), with limited mathematical and programming experience.

As discussed above, most of the theoretical models can simulate a shell that has a form resembling the actual shell in a realistic 3D geometry, based on shell ontogeny processes. In contrast, empirical morphometrics can only quantify and compare certain dimensions of actual shells. Clearly, the theoretical approach is better than the empirical approach in its accuracy of shell form quantification, because accurate morphological quantification is essential for functional, ecological and evolutionary studies of shell form. Below, we identify and discuss a few impediments that prevent empirical morphologists from adopting the theoretical approach in shell form quantification at the present and in the past.

First, the requirement of a computation resource was an impediment in the past. These theoretical models may only be implemented in a computation environment. As mentioned above, the advances of computation hardware in speed and 3D graphic technology have promoted the development of more complex theoretical shell models. For example, the current speed and storage of a desktop computer is at least four orders of magnitude greater than those used by Cortie [20] only two decades ago. Clearly, the computation hardware is no longer an impediment (e.g. [57]) for the application and development of theoretical shell models.

Notwithstanding the hardware development, programming skills are still a prerequisite for the implementation of theoretical models. Many of the early models that were published between the 1960s and 1990s, used third-generation programming languages such as Fortran and C++, which essentially lack of easily accessible graphic APIs. This situation has improved now that the simulation of theoretical shell models can be done in fourth-generation programming languages such as Mathematica [28,55,56,58] and MATLAB [2,32,59]. Most of these shell models were described with intensive mathematical notation, at least from a biologist’s point of view, in the publication; and some of these were published together with the information on algorithm implementation. However, the actual programming codes are rarely published together with the paper though they may be available from the authors upon request (but see [28,55,58]). Only two theoretical modelling software packages based on Raup’s model have a graphic user interface that is comparable to contemporary geometric morphometric software [36,58]. However, both of these software packages cannot be used for irregularly coiled shells. The rest of the modern theoretical models are far less approachable than the morphometric software for empirical morphologists. This is because those advanced theoretical models have not been delivered in a form that allowed empirical morphologists to have “hands-on experience” with them, without extensive mathematical literacy [57,60].

Second, theoretical shell models simulate the shell form based on the input of a set of parameters, which could be non-biological or/and biologically meaningful. Non-biological meaningful parameters are counter-intuitive for empirical morphologists because these parameters are not intrinsic shell traits. Nevertheless, many of these non-biological parameters are required for the model to fit the shell form schematically [61]. When the biological parameters do represent shell traits, they are often difficult to obtain accurately and directly from the actual shell because of the three-dimensional spiral geometry [12,14,19,36,61–65]. Since the development of theoretical shell models, almost all simulated shell models have been made by an ad hoc approach, where the parameters are chosen for the model and then the simulated shells are compared with the actual shells. In almost all cases, the correct parameters are chosen after a series of trial-and-error, and the parameters are selected when the form of the simulated shell matches the actual shell. Okamoto [12] suggested that this ad hoc approach based on pattern matching was easier than obtaining the parameters empirically from the shell.

Third, although the overall forms of the simulated shells resemble the actual shells, the simulated shell is not exactly the same as the actual shell [41,66]. For many models, its original parameters are not sufficient to simulate the shell form exactly [17,64]. These simulated general shell forms are adequate for theoretical morphologist interests in their exploration of general shell forms. However, the subtle features on a real shell or the subtle differences among different shell forms of real species that cannot be simulated by theoretical models may have significant functional implications that are important for empirical morphologists.

In brief, it is clear that the implementation of current theoretical shell models is less accessible to empirical shell morphologists. This has caused the utility of growth models for descriptive and discriminator purposes to have been underappreciated. Yet, empirical morphologists are using traditional and geometric morphometrics as a routine method for shell quantification.

### Popularity of traditional and geometric morphometrics in empirical studies

In addition to the impediments arising from the theoretical shell model itself that are limiting its popularity among empirical morphologists, the theoretical approach faces competition from geometric morphometric methodology. The popularisation of desktop computing that led to the flourishing of theoretical shell models in the late 1980s, also promoted the development of morphometric methods, such as Elliptical Fourier Analysis (EFA) and geometric morphometrics (GM). Rohlf and Archie [67] set a benchmark for the quantification of an organism’s form by EFA, which was improved from Kaesler and coworkers [68,69]. Rohlf and Slice [70] and Bookstein [71] developed a complete standard protocol for GM. In fact, geometric morphometrics was developed mainly by Bookstein [72,73] based on the idea of Thompson (see Chapter 17 in [74].

Soon after these pioneer papers, various software with Graphic User Interface (GUI) were developed for the application of EFA and GM ([75], see http://life.bio.sunysb.edu/morph/). In contrast to the application of theoretical shell models, an understanding of mathematics and programming languages is not a prerequisite for the user of these morphometric tools. Thus, EFA and GM have been well received by biologists, and have been adopted in the morphometric study of shell form. To our knowledge, GM was first applied in a shell morphometric study by Johnstone et al [50] when they placed the varix-suture intersection landmark along the spiral. On the other hand, EFA was first used by Costa et al. [76].

These geometric morphometric software packages have standard and interactive workflows that help empirical morphologists in every step of: obtaining morphometric data (e.g. placing landmark coordinates), analysing data (e.g. procrustes superimposition), statistical analysis (e.g. ANOVA, PCA), and visualising shape and shape changes (e.g. thin-plate spline, PCA plots). This has made geometric morphometrics approachable to empirical morphologists, who want to examine the similarities and differences among shell forms. However, geometric morphometrics is actually a statistic of shape that is calculated from Cartesian coordinate data from a sample of objects [75]. Hence, it is not an exact quantification of form and is not particularly suitable for comparison and quantification of shell form, for the following two reasons.

Geometric morphometrics can be practically useful when the shape comparisons are made among taxa or shells that are similar in shell form—usually in a narrow taxonomic range. However, Geometric Morphometrics that is strictly based on homologous landmarks would have little use for shape comparison among a wide range of taxa or shell forms. In most cases, 2D landmarks are chosen at the shell apex, suture, and aperture or whorl outline that can be identified from a 2D image that is taken in standard apertural view of a shell. This is especially the case in our study where great variation in shell form exists among the species within a very narrow taxonomic range. As reviewed in Liew et al. [77], these taxa are extremely hard to compare because of their unconformity in shell coiling regime and the fact that the typical aperture standard view cannot be applied to these shells, and hence it is not possible to obtain sufficient biologically homologous landmarks.

GM was formalised and developed by Bookstein [72,73] based on the conceptual idea of Thompson (see Chapter 17 in [74]. In the same publication, Thompson did not use the conceptual GM, but used a logarithmic spiral approach to compare shells (see Chapter 11 in [74]. Furthermore, shells were also not included in Bookstein [71] despite various examples of different organisms to show the effectiveness of GM in shape comparative analysis. Biologically meaningful homologous landmarks are absent from some of the shells.

Second, the results of separate, independent studies of shell forms cannot be integrated, even though these studies use the same GM method. Statistical analysis of the Cartesian coordinate data that abstractly represent the shell form is adequate in quantifying the variation of a shell within a context of other shells that are included in a single study or within similar taxa where similar landmarks are obtained. However, the raw coordinate data and analysed shape variation from a study are incomparable and incompatible with the data from other studies [78]. For example, the raw data (coordinates) from two studies cannot be combined if they use different landmarks and the shape variables (e.g. PCA scores) from a study cannot be compared and analysed together with other studies.

Despite the fact that geometric morphometrics has been widely used by empirical morphologists, it is not an ideal tool in the quantification of shell form for the reasons given above. Hence, it is important to return to the core of the question: what do biologists want to learn from the study of shell form? Clearly, in addition to quantitatively compare shell forms, biologists want to know more about the general characteristics and physical properties of the shell form that are key elements in gaining insight into functional and ecological aspects of the shell [79]. However, functional and ecological aspects of shell form can only be determined if the shell form can be exactly quantified.

### Using 3D technology to quantify shell form based on aperture ontogeny profiles

In this paper, we propose an interactive approach to the quantification and analysis of shell forms based on state of the art 3D technology and by integrating the theoretical principles of shell modelling and the empirical principles of morphometric data handling. There are no theoretical models that can simulate all existing shell forms. However, the theoretical background of the theoretical models is biologically sound—simulating the shell form by simulating the shell ontogenetic process. On the basis of this shell-ontogenesis principle, we used state-of-the-art X-ray microtomography (micro-CT scan) and 3D modelling software to obtain a series of shell aperture changes from the shell in an interactive workflow that is similar to empirical morphometric analysis. All our procedures were implemented by using open source and free software with the exception of 3D scanning instrumentation and software.

First, a series of shell aperture outlines were digitised directly from the reconstructed 3D shell model obtained from micro-CT scanning by using open-source 3D-modelling software—Blender ver. 2.63 (www.blender.org). Then, the growth trajectory and form of the shell aperture outline were quantified and extracted with our custom scripts that run in Blender through its embedded open-source Python interpreter (http://www.python.org/). The changes of aperture size and shape, and aperture growth trajectory in terms of curvature and torsion along the shell ontogeny axis length were obtained (hereafter “aperture ontogeny profiles”, see [80]). The final aperture ontogeny profiles are in a form of multivariate time series data, which consist of a number of instances (i.e. number of quantified apertures that depends on the length of the whorled shell tube) and attributes that represent the growth trajectories, aperture form, and size. These aperture ontogeny profiles can be plotted when each shell is examined individually. In addition, the differences between shells can be assessed quantitatively by calculating the dissimilarity of aperture ontogeny profiles among shells. Furthermore, the dissimilarity matrix can be used to plot the dendrogram. A detailed step-by-step manual and a video tutorial are available as S1 Protocol and S1 Movie.

Finally, we discuss some possible applications and implications of these shell form quantification methods in theoretical morphology, functional morphology, taxonomy and shell shape evolutionary studies.

## Materials and Methods

### Ethics Statement

Specimens were collected in Malaysia with permissions from the Economic Planning Unit, Malaysia (UPE: 40/200/19/2524).

### Scanning instrumentation

A micro-CT scanner (SkyScan, model 1172, Aartselaar, Belgium) and its accompanying software, NRecon ver. 1.6.6.0 (Skyscan©) and CT Analyser ver. 1.12.0.0 (Skyscan©), were used to generate digital shell 3D models from the actual shell specimens.

### Computation software and hardware

Various commercial 3D modelling and statistical software exist for visualising, manipulating, and understanding morphology, such as Amira^®^ (Visage Imaging Inc., San Diego, CA) and Autodesk Maya (San Rafael, CA) (reviewed by [81]). However, in this study, we used only two open-source 3D data modelling and processing software packages, namely Blender ver. 2.63 (www.blender.org) and Meshlab ver. 1.3.2 ([82], http://meshlab.sourceforge.net/). Both have been used in biology to visualise and model morphology (for Meshlab: [83–85]; for Blender: [86–93]). However, these programs have not been used to their full extent in morphological quantification and analysis of 3D data for organisms. For quantification of morphology, we used the open-source Python interpreter ver. 3.2 that is embedded in Blender 2.63. In addition, we also used an extension to the Python programming language—NumPy [94] which consists of high-level mathematical functions.

All the morphological data were explored and analysed with the statistical open source programming language R version 3.0.1 [95] in the environment of RStudio [96]. We installed two additional packages in R, namely, "lattice": Lattice Graphics [97], and "pdc": Permutation Distribution Clustering [98,99].

All the computation analyses were carried out with a regular laptop computer with the following specifications: Intel^®^Core^™^i7-3612QM @ 2.1GHz, 8 GB memory (RAM), NVIDIA^®^ GeForce GT 630M with 2GB memory.

### Procedures

#### 1. Obtaining digital 3D models from actual shells

The scan conditions were as follows: voltage– 80kV or 100kV; pixel– 1336 rows × 2000 columns; camera binning– 2 × 2; image pixel size– 3–6 μm; rotation step– 0.4° or 0.5°; and rotation– 360°. Next, the volume reconstruction on the acquired images was done in NRecon. The images were aligned to the reference scan and reconstruction was done on the following settings: beam hardening correction– 100%; reconstruction angular range– 360 degrees; minimum and maximum for CS to image conversion (dynamic range)–ca. 0.12 and ca. 20.0; and result file type—BMP. Finally, 3D models were created from the reconstruction images in CT Analyser with the following setting: binary image index– 1 to 255 or 70 to 255; and were saved as digital polygon mesh object (*.PLY format).

#### 2. Pre-processing digital shell models

The 3D models were then simplified by quadric edge collapse decimation implemented in MeshLab [82] to reduce computation requirements. The raw polygon mesh shells in PLY format have millions of faces and a file size between 20 to 80 Mbytes. Thus, we reduced the number of faces for all model to 200,000–300,000 faces, which range between 3 and 6 Mbytes in file size. In addition, for the sake of convenience during the retopology processes, all 3D models were repositioned so that the shell protoconch columella was parallel with the z-axis. This was done by using manipulator tools in MeshLab.

#### 3. Creating reference: Tracing aperture outlines and ontogeny axis from shell models

(S1 Movie: from 00:40 until 22:00 of the video). The digital shell 3D model in PLY format consists of 3D Cartesian coordinate vertices in which each of the three vertices constitutes a triangular face, and all faces are connected through a complex network. In order words, these vertices and faces are not biologically meaningful structures, and it is not possible to extract aperture outlines data directly from a raw PLY digital shell model. Monnet et al. [100], for example, attempted to extract aperture outline automatically from a digital 3D model by making a plane cross-sectioning of the shell model, but its outlines do not reflect the form of the actual aperture outlines. Hence, we retopologised the raw 3D mesh models according to the aperture ontogeny for later data extraction.

We used Blender, which is more flexible than the commercial software used by Monnet et al. [100]. For the sake of convenience, we describe the following workflow, including the tools or the function (e.g. “Import PLY”) which can be called after hitting the SPACE bar while in the Blender environment. However, this workflow may be modified by the user.

**Fig 1:**
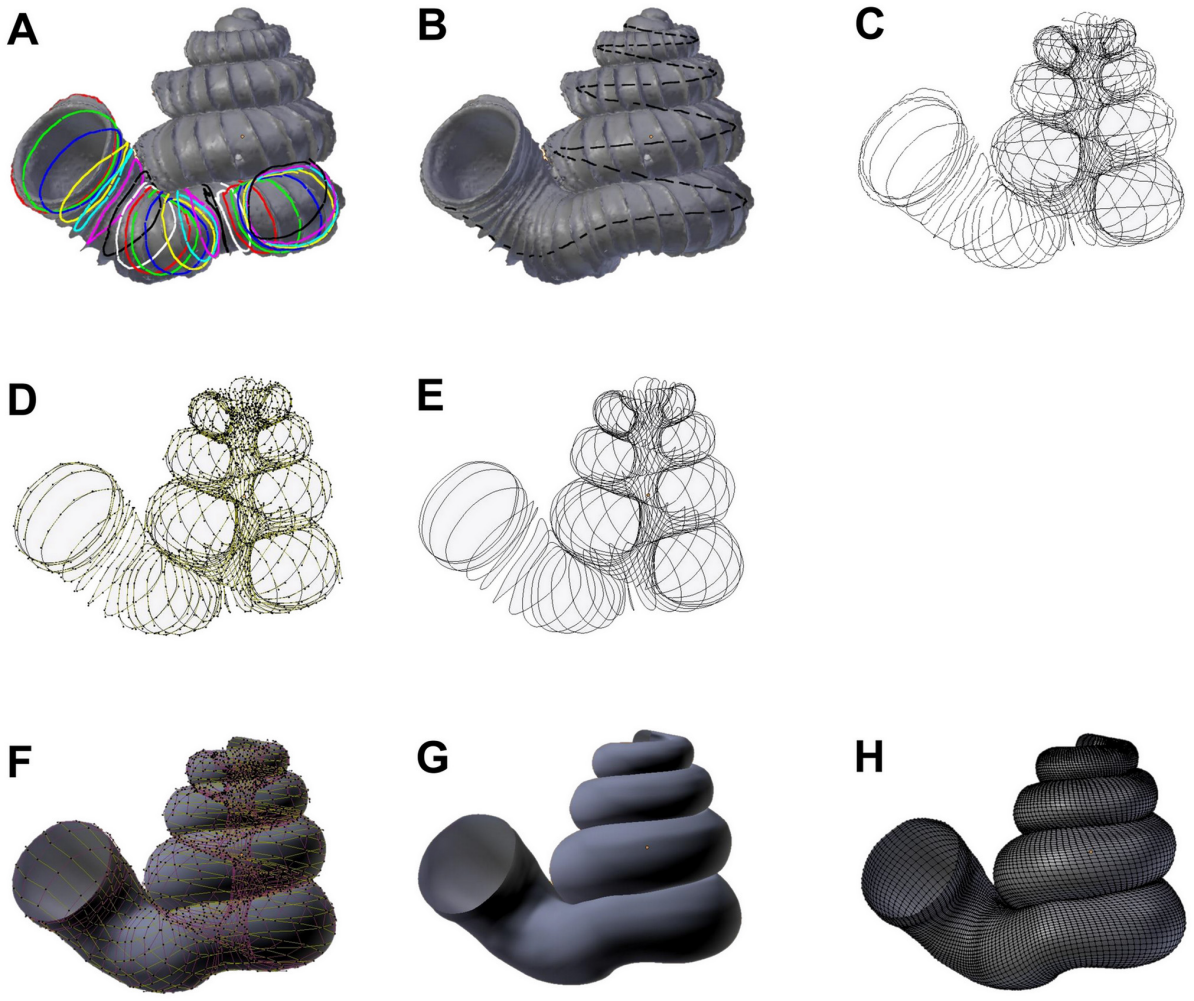
Procedures to generate a retopologised shell based on the aperture ontogeny from a shell by using Blender software. (A) Procedure 3—Creating reference: Tracing aperture from shell model. (B) Procedure 3—Creating reference: Tracing ontogeny axis. (C) Procedure 3 –both traced aperture outline and ontogeny axis were converted to Bezier curves. (D) Procedure 4 –Retopologising aperture outlines from the reference by using NURBS circles in EDIT mode. (E) Retopologised aperture outlines. (F) Procedure 4 –Generating retopologised shell surface models from NURBS circles in EDIT mode. (G) Final retopologised NURBS surface shell model. (H) Retopologised 3D shell mesh converted from retopologised NURBS surface shell model.

To begin, we imported a PLY shell model into the Blender environment (“Import PLY”). Then, we resized the model 1000 × (“Resize”) so that the scale of 1 Blender unit was equal to 1 mm. After that, we examined the traces of aperture outlines (i.e. growth lines, ribs, spines) (Fig 1A) and ontogeny axis (i.e. spiral striation, ridges, colour lines) (Fig 1B) of the actual shells. However, it is not possible to trace apertures from the shell protoconch because the protoconch is an embryonic shell that may not grow accretionarily and usually has no growth lines. In many cases, the aperture of the overlapping whorls cannot be traced from the outer shell wall. One of the ways to deal with this situation is to trace the aperture at the inner shell wall and the obscured aperture outline can then be inferred by studying conspecific juvenile specimens (see video tutorial 05:00–08:00 of S1 Movie). It does not really matter whether the aperture outline was traced from outside or inside. After it was traced from the inside, the subsequent retopologising stage would need take into consideration the shell thickness of the overlapping whorl.

After these aperture traits were identified, we selected the 3D model (by clicking “right mouse button”), and traced all these traits on the surface of the raw 3D mesh model in Blender by using the “Grease Pen Draw” tool. After that, the grease pen traced aperture traits were converted to Bezier curves with “Convert Grease Pencil” (Fig 1C). We would like to emphasise that this is the most critical step that determines the efficiency of this shell quantification method. Thus, the key lies in the good understanding of the way the aperture is structured, which is essential to trace the aperture outlines accurately. However, the orientation of the shell when the aperture is digitalised would not influence the aperture ontogeny data.

#### 4. Retopologising aperture outlines from the reference and generating retopologised shell models

(S1 Movie: from 22:01 until 53:00 of the video; and File 4). For each shell, we created a set of new Non Uniform Rational B-Splines (NURBS) surface circles (“Add Surface Circle”) and modified these (“Toggle Editmode”) according to the aperture outlines. We created a 16 points NURBS surface circle and aligned the circle to the aperture outline by translation (“Translate”), rotation (“Rotate”), and resizing (“Resize”) (Fig 1D). After the NURBS surface circle was generally aligned, each of the 16 points of the NURBS surface circle were selected and adjusted by translation (“G”) one by one, so that the outline of the NURBS surface circle was exactly the same as the aperture outline. At the same time, the second point of the NURBS surface circle was aligned to the ontogeny axis (Fig 1B and 1C). In the case of the shells that we used in this study, which have a relatively simple, almost circular aperture, 16 points is sufficient to capture the aperture’s outline. In the case of more complex aperture shapes, a greater number of points of NURBS surface circle may be required to capture the aperture’s outline. In any case, the number of chosen points will not affect the final surface model that will be generated by these surface circles, as long as the aperture shape is properly represented by the NURBS surface circles.

After the first aperture outline was retopologised as a NURBS surface circle, the NURBS surface circle was selected by using a python script (S2 Text), duplicated (“Duplicate Objects”) and aligned to the next aperture outline as the previous one. This step was repeated until all the aperture outlines were retopologised into NURBS surface circles (Fig 1D and 1E). Then the shell surface was created in the form of a NURBS surface based on the digitised aperture NURBS surface circle (“(De)select All” and “Make Segment” in “Toggle Editmode”) (Fig 1F and 1G). Lastly, we made the surface meet the end points in U direction and increased the surface subdivision per segment (resolution U = 8) through the properties menu of the object (Properties (Editor types)>Object Data>Active Spline).

After that, we converted the NURBS surface 3D model into a 3D Mesh model that consists of vertices, edges, and faces (“Convert to”—“Mesh from Curve/Meta/Surf/Text”). The final retopologised 3D shell Mesh consists of X number of apertures outlines and each aperture outline has Y number of vertices and then a total of X*Y vertices. Each of the vertices is connected to four other nearest vertices with edges to form a wireframe shell and face (Fig 1H).

It is important to note that the NURBS surface circle is defined by a mathematic formula which does not imply any biology perspective of the shell. We choose NURBS surface circle because the 3D aperture outline form can be digitalised by a small number of control points and shell surface can be recreated by NURBS surface based on the digitised aperture NURBS surface circle. The final 3D polygon mesh model is more simplified than the raw PLY 3D model and each of its vertex data resemble the actual accretionary process of the shell (Fig 1A and 1H).

#### 5. Quantifying aperture growth trajectory

(S1 Movie: from 53:01 until 56:00 of the video). The aperture ontogeny profiles were quantified as described in Liew et al. [80] with slight modifications where both aperture growth trajectory and aperture form were quantified directly from the retopologised 3D shell model. This aperture growth trajectory was quantified as a spatial curve, which is the ontogeny axis as represented by a series of first points of the aperture outlines. We estimated two differential geometry parameters, namely, curvature (κ) torsion (τ), and ontogeny axis length for all apertures [12,29]. The local curvature and torsion, and accumulative ontogeny axis length were estimated from the aperture points along the growth trajectory by using weighted least-squares fitting and local arc length approximation [101]. All the calculations were done with a custom-written Python script which can be implemented in Python interpreter in the Blender ver. 2.63 environment. The whole workflow was: (1) selecting the retopologised 3D shell Mesh (by clicking “right mouse button”), (2) input parameters for number of sample points “q = ##” in the python script, and (3) paste the script into the Python interpreter (S1 Text). The final outputs with torsion, curvature and ontogeny axis reference for each aperture were saved as CSV files.

We found a convergence issue in curvature and torsion estimators (see also [36,101]). The accuracy of the curvature and torsion estimates depends on the number and density of the vertices in the ontogeny axis (i.e. number of aperture outlines), and the number of sample points. Nevertheless, different numbers of sample points can be adjusted until good (i.e. converged) curvature and torsion estimates are obtained. We used 10% of the total points as number of sample points of the ontogeny axis, which gave reasonably good estimates for curvature and torsion.

Notwithstanding the algorithm issue, the curvature and torsion estimators are informative in describing the shell spiral geometry growth trajectory. Curvature is always larger or equal to zero (κ ≥ 0). When κ = 0, the spatial curve is a straight line; the larger the curvature, the smaller the radius of curvature (1/ κ), and thus the more tightly coiled the spatial curve. On the other hand, the torsion estimator can be zero or take either negative or positive values (- ∞ ≤ τ ≤ ∞). When τ = 0, the spatial curve lies completely in one plane (e.g. a flat planispiral shell), negative torsion values correspond to left-handed coiling and to right-handed coiling for positive torsion values; the larger the torsion, the smaller the radius of torsion (1/ τ), and thus the taller the spiral.

#### 6. Quantifying aperture form

(S1 Movie: from 53:01 until 56:00 of the video). We quantified the aperture outline sizes as perimeter and form as normalised Elliptic Fourier coefficients (normalised EFA) by using a custom-written Python script which can be implemented Python interpreter embedded in the Blender environment. The workflow was (1) selecting the retopologised 3D shell mesh (by clicking “right mouse button”), (2) input parameters for “number_of_points_for_each_aperture = ##” in the python script, and (3) paste the script into the Python interpreter of Blender (S1 Text). The final outputs were saved as CSV files.

Aperture outline perimeter was estimated from the sum of lengths (mm) for all the edges that are connecting the vertices (hereafter “aperture size”). For aperture form analysis, we used 3D normalised EFA algorithms [102] and implemented these in the custom python script. Although many algorithms exist for describing and quantifying the form of a closed outline [103], we used EFA because it is robust to unequally spaced points, can be normalised for size and orientation, and can capture complex outline form with a small number of harmonics [67,102]. In this study, we used five harmonics, each with six coefficients which were sufficient to capture the diverse aperture shapes of our shells. For quantification of apertures shape that are invariant to size and rotation, we normalised EFA of aperture outlines for orientation and size. If needed for comparison with other studies, the normalised EFA can be repeated for the same dataset with higher or lower numbers of harmonics.

After normalisation, we ran principal components analysis (PCA) to summarise the 30 normalised Fourier coefficients as principal components scores (hereafter “aperture shape scores”). After that, we selected the major principal components (explaining > 90% of the variance) for further analysis. The aperture shape scores of each selected principal component were plotted and analysed against the ontogeny axis.

#### 7. Visualising aperture form and trajectory changes along the shell ontogeny

For exploration of data, we used a graphical technique for representing aperture ontogeny profile changes along the shell ontogeny. For each shell, we made a vertical four-panels scatter plot in which each of the four variables (namely, curvature, torsion, aperture size, and the first principal component aperture shape score) were plotted against the ontogeny axis. When necessary, the second and third principal component aperture shape scores were also included. In addition, the axis of each variable was rescaled so that it was the same for the same variable of all shells. After standardisation of the axis, the aperture ontogeny profiles of several shells could be quantitatively compared side by side.

#### 8. Quantitative comparison between shell forms

In addition to the qualitative comparison between shells forms as described above, the dissimilarity between different shells can be analysed quantitatively. We used Permutation Distribution Clustering (PDC) which finds similarities in a time series dataset [98,99]. PDC can be used for the analysis of the changes in a variable along shell ontogeny between different shells (i.e. two-dimensional dataset: number of shells × number of apertures) and multiple variable changes between shells (i.e. three-dimensional dataset: number of shells × number of variables × number of apertures). We applied the most recent analysis developed by Brandmaier [98,99] because it has an R package that can be applied and can calculate the trend similarity. That said, the same data can always be analysed by other algorithms that may become available in the future.

Although PDC is robust to the length differences between datasets, our preliminary analysis showed that the PDC output would be biased when there was a great (around two-fold) length difference in the total ontogeny axis length. As we compared the entire ontogeny profiles (i.e. right after the protoconch until the final aperture) among the shells, larger shells would have a longer ontogeny axis. Thus, we resampled the ontogeny profiles (100%) at each 2% of the ontogeny axis of each of the shells. Hence, we standardised the data as in procedure 7, but dividing the ontogeny axis of each shell into 50 equal length intervals and obtained the ontogeny profiles data at these 50 points along the ontogeny axis. This standardisation procedure allows comparison of trends in variable changes along the shell ontogeny. In addition to the shape comparison, we obtained the shell size in terms of volume by using “Volume” function in Blender after the 3D shell model was closed at both ends by creating faces “Make edge/Face”) on selected apertures at both end (“Loop Select”) in EDIT mode.

The aperture ontogeny profiles of all shells were combined into a three-dimensional data matrix consisting of n shells × four variables × 50 aperture data points. We ran five PDCs, each for the five data matrices with: 1) all four variables, 2) torsion, 3) curvature, 4) aperture size, and 5) aperture shape scores. The parameter settings for the PDC analysis were as follows: embedding dimension = 5; time-delay of the embedding = 1; divergence measure between discrete distributions = symmetric alpha divergence; and hierarchical clustering linkage method = single. The dissimilarity distances between shells were used to produce the dendrogram. PDC analysis was performed with the “pdc” library [99] in R version 3.0.1 [95] (S3 Text).

### Worked example: Comparative analysis of *Opisthostoma* and *Plectostoma* species shell form and simulated shell form

We evaluated the above-described shell form quantification method by using the shells of *Opisthostoma* and *Plectostoma*, which exhibit a great variability in shell form. Some of the species follow a regular coiling regime whereas others deviate from regular coiling in various degrees. It remains a challenging task to quantify and compare these shell forms among species, either by using traditional or geometric morphometrics, because a standard aperture view for the irregular and open coiled shells cannot be determined.

**Fig 2:**
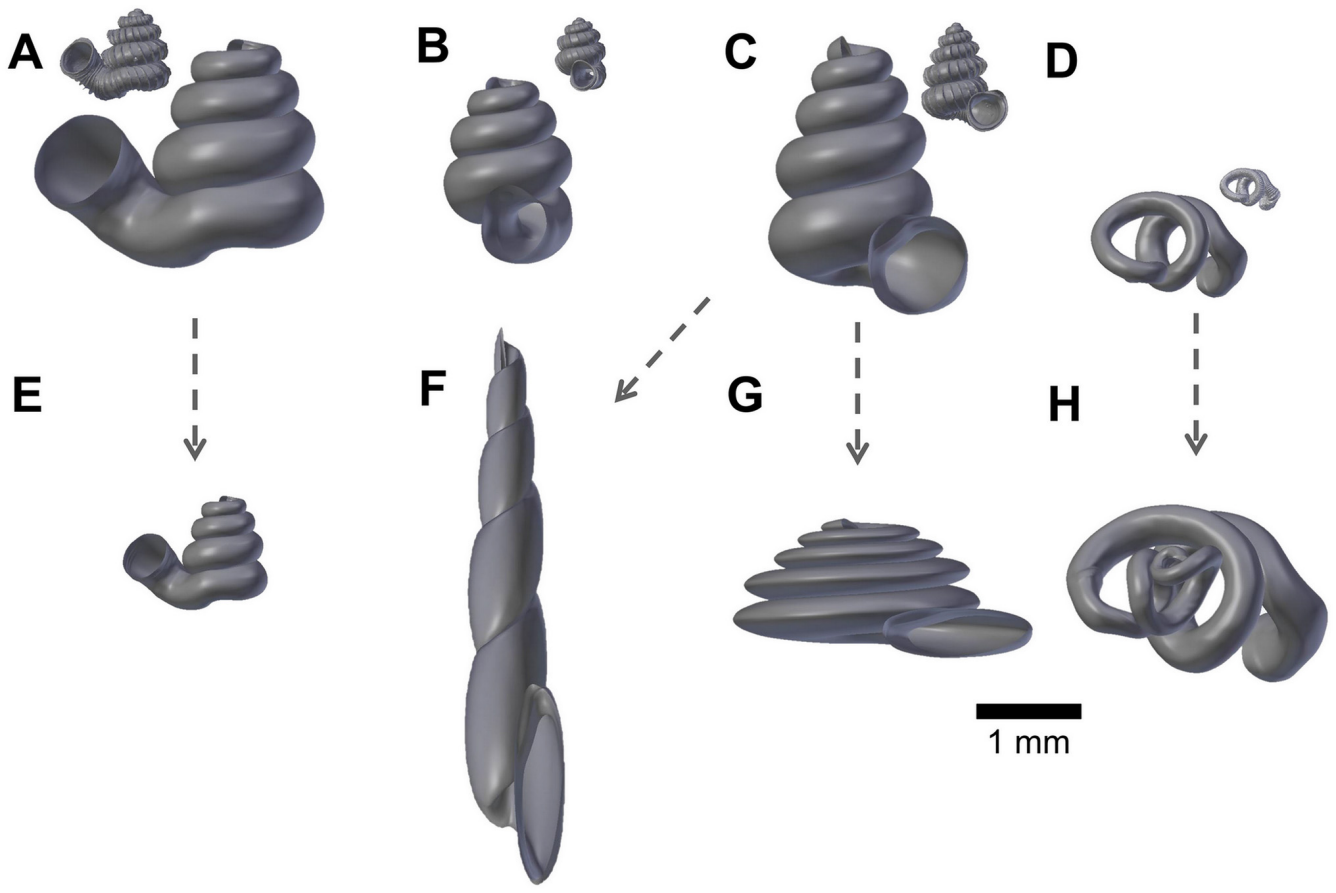
Retopologised shell 3D models obtained by repotologising real shells (A–D) and by transformation of retopologised shells (E–H). (A) Shell of *Plectostoma laidlawi* Sykes 1902. (B) Shell of *Plectostoma crassipupa* van Benthem Jutting, 1952. (C) Shell of *Plectostoma christae* Maassen 2001. (D) Shell of *Opisthostoma vermiculum* Clements and Vermeulen, 2008. (E) *Plectostoma laidlawi* shell that was resized by one-half and with slight modification of the last aperture size. (F) *Plectostoma christae* shell that was reshaped into an elongated form by reducing the model size (linear dimension) by one-half along the x and y axes, and by doubling the size along the z axis. (G) *Plectostoma christae* shell that was reshaped into a depressed form by increasing by 1.5 the model size along the x and y axes, and by reducing the size by one-half along the z axis. (H) *Opisthostoma vermiculum* shell that consists of one *Opisthostoma vermiculum* original 3D model of which the aperture was connected to a second, enlarged, *Opisthostoma vermiculum*.

We selected four species, namely, *Plectostoma laidlawi* Skyes 1902 (Fig 2A), *Plectostoma crassipupa* van Benthem Jutting, 1952 (Fig 2B), *Plectostoma christae* Maassen 2001 (Fig 2C), and *Opisthostoma vermiculum* Clements and Vermeulen, 2008 (Fig 2D), for which the shell forms are, respectively: regularly coiled, slight distortion of the last whorl, strong distortion of the last whorl, and complete distortion of most of the whorls. Despite the narrow taxonomic range of the selected species, the range of shell forms of these four species do cover a very large diversity of shell form. We retopologised these four shells by following the procedures 1 to 4 (S1 Dataset).

**Fig 3:**
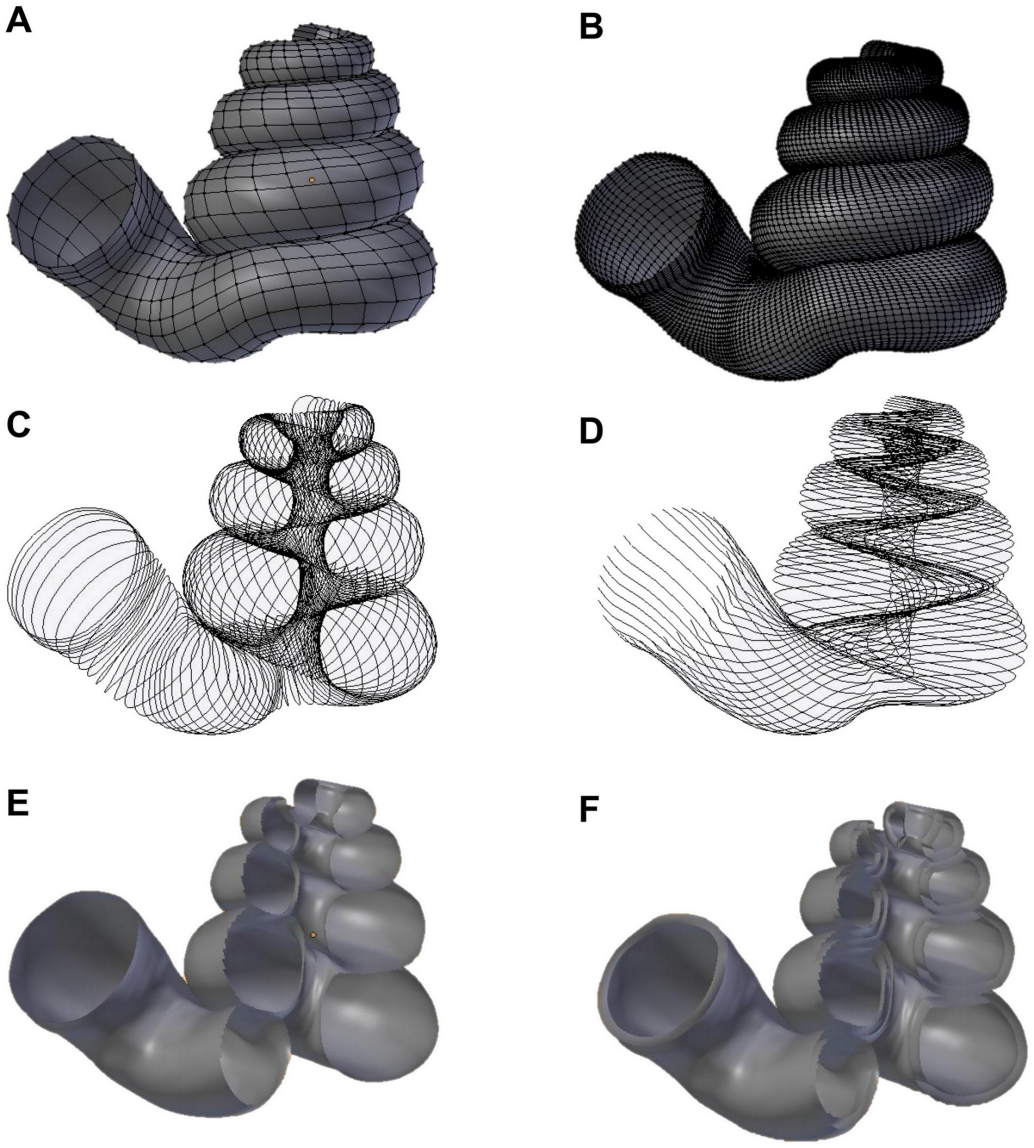
Different data types that could be obtained directly from a 3D shell model that was retopologised on the basis of the aperture ontogeny and which can be used in theoretical modeling (A–D) and functional morphology studies (E–F). (A) Aperture maps (*sensu* Rice, 1998) or growth vector maps (*sensu* Urdy et al., 2010). (B) same as (A), but the data can be obtained in a greater resolution. (C) Aperture outlines data for generating curve models. (D) Multiple ontogeny axes for helicospiral models. (E) Simple 3D surface shell model with no thickness. (F) 3D surface shell model with added thickness.

In addition to the four retopologised 3D shell models, we manually created another four shell models by transforming three out of the four retopologised NURBS surface 3D shell models by using the “Transform” function in Blender. These models are: 1) *Plectostoma laidlawi* that was resized to half the original size and given slight modification of the aperture size (Fig 2E); 2) *Plectostoma christae* that was reshaped into an elongated form by reducing the model size (linear dimension) to one-half along the x and y axes, and by doubling the size along the z axis (Fig 2F); 3) *Plectostoma christae* that was reshaped into a depressed form by multiplying by 1.5 the model size along the x and y axes, and by reducing to one-half along the z axis (Fig 2G); and 4) *Opisthostoma vermiculum* that consists of one *Opisthostoma vermiculum* original 3D model of which we connected the aperture to another, enlarged, *Opisthostoma vermiculum* (Fig 2H). Finally, we analysed all these eight shell models by following the procedures 5 to 8.

## Results and Discussion

### Retopologied 3D shell models

All the final retopologised 3D shell models can be found in PLY ASCII mesh format (S2–S9 Datasets), with the raw data as a list of vertices, followed by a list of polygons, which can be accessed directly without the need of any 3D software. Each vertex is represented by x, y, z coordinates. Each polygon face consists of four vertices. This simplified yet biologically informative 3D mesh shell model allows the quantification of aperture form and growth trajectory. Moreover, the 3D shell models and their raw vertices data could potentially be used in studies of functional morphology and theoretical modelling of shell form, respectively.

Malacologists have been focusing on empirical shell morphological data, from which the functional, ecological and evolutionary aspects were then extracted. The physical properties were then determined by its form (e.g. [55,56]). By using the 3D models, the shell properties and function can be analysed *in silico*. For example, the thickness of the shell can be added to the 3D shell model (Fig 3E and 3F) in order to obtain the shell material’s volume, the shell’s inner volume, its inner and outer surface area, and centre of gravity. We used the “build” function of the software, which can only “solidify” the model by uniform thickness. However, if necessary, it is possible to write a custom Python script to add the desired thickness to the shell. Quantification of shell properties may then be done by using the geometry approach in Meshlab or Blender, as compared to the pre-3D era where mathematical descriptions of the shell form were required [3,104,105]. Furthermore, it is possible to convert the 3D models to a 3D finite element (FE) model, of which the physical properties (e.g. strength) can be tested (e.g. [32]).

In addition to the potential use of 3D shell models in functional morphology, the coordinate data of the vertices of 3D shell models could be used directly by theoretical morphologists (see Figure 1 in [2]). For example, these data can be extracted in different formats that fit the data requirement of different types of theoretical shell models, namely, generating curve models using a fixed reference frame or moving reference frame (Fig 3C), helicospiral or multivector helicospiral models using a fixed reference frame (Fig 3A, 3B and 3D) or growth vector models using a moving reference frame (Fig 3A and 3B).

The retopologising of the aperture ontogeny from a raw 3D shell model (procedures 1 to 4) is a time-consuming and tedious process compared with traditional and geometric morphometrics. There are no differences in the time required for data analysis between GM and our method. The only time differences are in the data acquisition. In our experience, two to three days are needed to collect the aperture data from the shell. For example, the four shell models were created by retopologising between 73 and 96 separate apertures (ca. 1500 points for 90 apertures). From the viewpoint of short-term cost-benefit balance, this may be seen as a waste of time, because GM requires not more than a few dozen points for each shell, which can generate the shape variables for a study, even though these points are not comparable to other points of other shells or other studies. However, in the long run, it is a good time investment, since it will allow the understanding of shell function, growth, and evolution, as the same set of data is obtained from different shell forms and can be accumulated and analysed together. Moreover, as with all newly-developed techniques, improvements in efficiency and automation are possible and may remove these impediments in the future (e.g. [36]).

### Comparing shell form from the view of shell ontogeny

**Fig 4:**
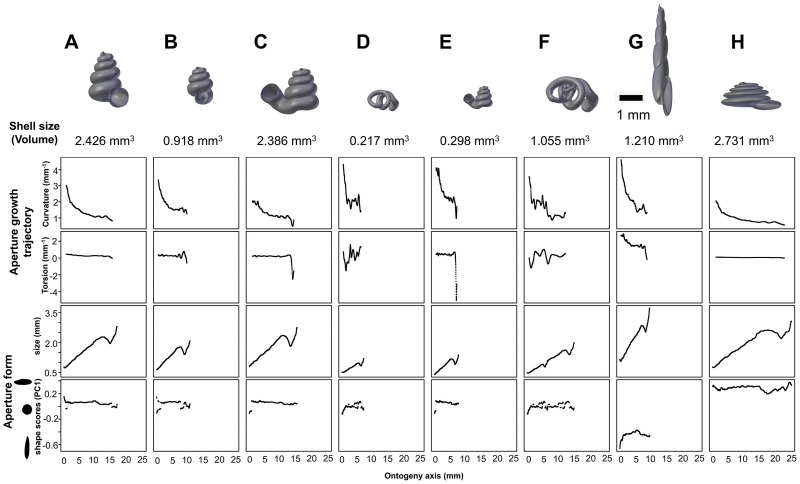
Shell size (volume) and aperture ontogeny profiles in terms of aperture growth trajectory (curvature and torsion) and aperture form (size and shape) of eight shells. (A) Shell of *Plectostoma laidlawi* Sykes 1902. (B) Shell of *Plectostoma crassipupa* van Benthem Jutting, 1952. (C) Shell of *Plectostoma christae* Maassen 2001. (D) Shell of *Opisthostoma vermiculum* Clements and Vermeulen, 2008. (E) *Plectostoma laidlawi* shell that was resized by one-half and with slight modification of the last aperture size. (F) *Plectostoma christae* shell that was reshaped into an elongated form by reducing the model size (linear dimension) by one-half along the x and y axes, and by doubling the size along the z axis. (G) *Plectostoma christae* shell that was reshaped into a depressed form by increasing by 1.5 of the model size along the x and y axes, and by reducing the size by one-half along the z axis. (H) *Opisthostoma vermiculum* shell that consists of one *Opisthostoma vermiculum* original 3D model of which the aperture was connected to a second enlarged *Opisthostoma vermiculum*.

Fig 4 gives an overview of the aperture ontogeny profile and shell volume for each species. The curvature, torsion perimeter, and ontogeny axis are represented by true numerical values with the unit of mm^-1^ and mm, and thus can be interpreted directly. In contrast, the aperture shape scores are just statistics of Fourier coefficients and are not the absolute quantification of aperture shape. The PCA score of an aperture shape depends on the shape of other aperture outlines and thus it might change whenever other aperture outlines are added into the analysis. Nevertheless, the aperture scores will stabilise as data of more shells become available and when most of the extreme aperture forms are included. In this study, the first principal component explained 92% of the total variance; the second and third principal component explained only 3% or 1% of the total variance. We showed that the shell form can be represented by the ontogeny changes of the aperture growth trajectory in terms of curvature and torsion, and aperture form, in terms of perimeter and shape.

**Fig 5:**
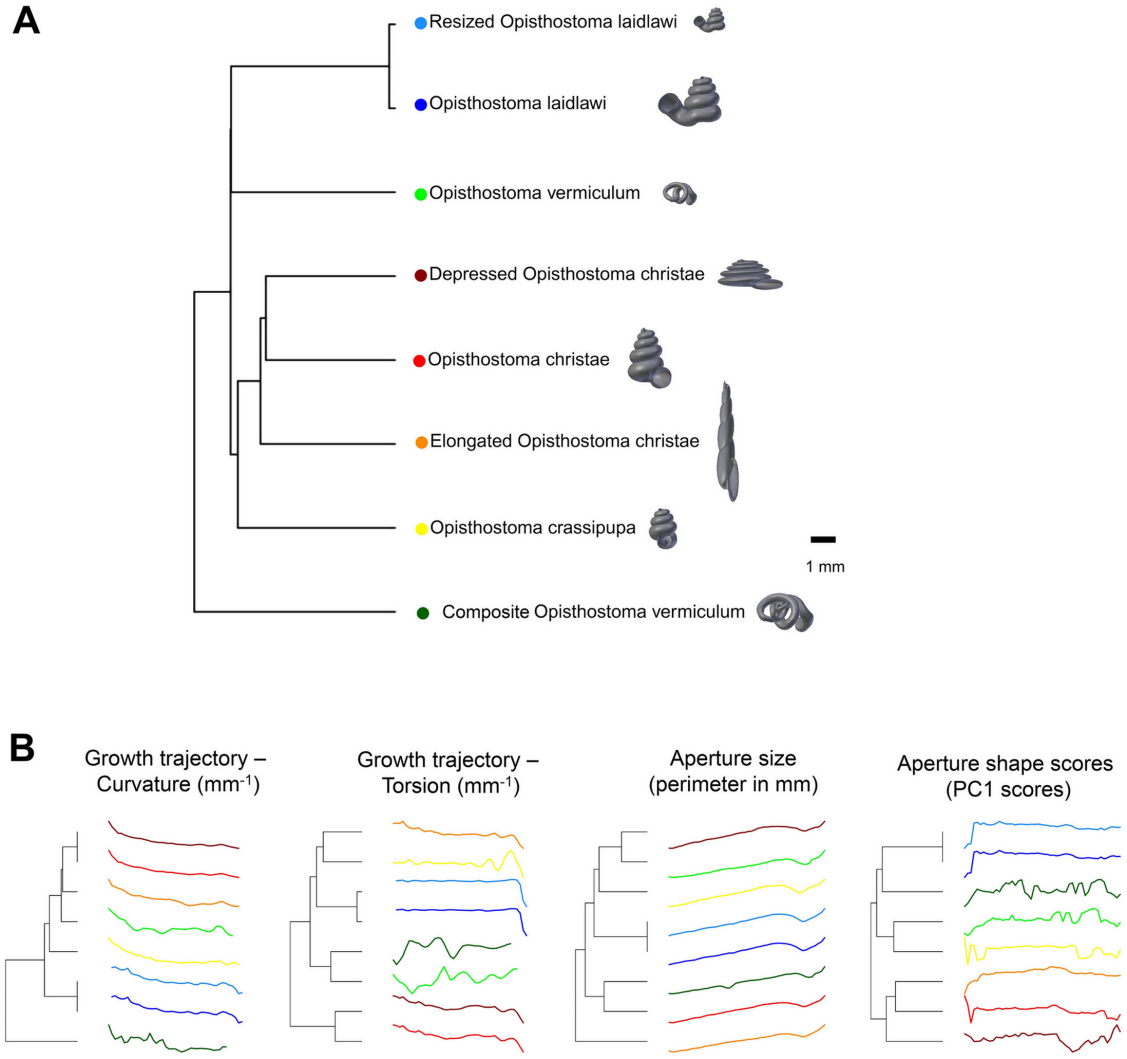
Dendrogram from permutation distribution clustering of the aperture ontogeny profiles of eight shells. (A) Dendrogram from permutation distribution clustering of the four aperture ontogeny profiles, namely, curvature, torsion, aperture size, and aperture shape scores, of eight shells. (B) Four dendrograms from permutation distribution clustering of eight shells, which each for the four aperture ontogeny profiles, namely, curvature, torsion, aperture size, and aperture shape scores.

Our first example evaluates this method in illustrating the differences between two shells that have the same shape but differ in shell size—the half-size *Plectostoma laidlawi* (Fig 4E) shell and the original *Plectostoma laidlawi* shell (Fig 4C). As revealed by their aperture ontogeny profiles, the size difference between the two shells has had an effect on the curvature, torsion, ontogeny axis length and aperture size. For the resized *Plectostoma laidlawi* shell, the values of curvature and torsion are twice as large as for the original, whereas the ontogeny axis length and aperture size are only half those of the original shell. The overall trends in the changes of these variables along the ontogeny axis are comparable between these two shells (Fig 5B).

Another example shows the ontogeny profiles of three shells, namely, the elongated (Fig 4G), depressed (Fig 4H), and original (Fig 4A) versions of the *Plectostoma christae* shell. Comparison of aperture profiles among these show the most obvious discrepancies in greater torsion values for the elongated shell, which change in a more dramatic trend along the shell ontogeny. In addition, each of the three shells has its unique aperture shape scores, though there are no big discrepancies in the aperture size. The differences in ontogeny axis length, curvature and torsion are related to the differences of the aperture shape statistics among the three shells. However, our small dataset with only three shells is not sufficient for thorough disentangling of the interplay between aperture size, shape, and growth trajectory in relation to the shell form.

Our last example is the comparison between the original (Fig 4D) and the composite (Fig 4F) *Opisthostoma vermiculum* shell. It is clear that our method has high sensitivity and robustness in the analysis of such bizarre shell forms. As shown in Fig 4F, the start of the aperture ontogeny profile of the composite shell was the same as for the original shell (Fig 4D). In addition, the later parts of the ontogeny profile trends are still comparable to the first part, but different in value because of the scalar effect.

As we have shown in Fig 4, the shell forms can be explored and compared qualitatively on the basis of aperture ontogeny profiles. Users might need some training in the interpretation of the plots because they are different from both linear dimension measurement plots and geometric morphometric shape coordinate plots. Our evaluation suggested that the data visualisation method is sensitive and robust in capturing the aperture ontogeny profile for any shell form and thus make the qualitative comparison across gastropod taxa and studies possible.

This method could be applied in malacological taxonomy because its core business is the description of shell form. Despite hundreds of years of taxonomic history of shells, there has been little change in the way shell form is being described. For example, shell from is usually described in terms of linear dimensions: shell width and height; number of whorls; shell shape—flat, depressed, globose, conical, or elongated; whorls shape—from flat to convex. Here, we suggest that the aperture ontogeny profiles would be a great supplement to the classical approach to shell description. For example: (1) the size of the shell (its volume) depends on the ontogeny axis length and aperture size; (2) the shell shape depends on the growth trajectory in terms of curvature and torsion; (3) the shape of the whorls depends on the shape of the aperture (Fig 4). In our case of the four shells (Fig 2A–2D), it is clear that aperture size of each shell is constricted at roughly the same part of the respective shell ontogeny, namely between 70% and 85%, regardless of the dissimilar shell sizes and shapes (Fig 4A–4D). In fact, these aperture size decreases during ontogeny are in accordance with the shell constriction, one of the shell characters that have been used in the taxonomy of the genera *Opisthostoma* and *Plectostoma* [77, 106]. However, the shell constriction has not been quantified previously, and we show that it could also be an important developmental homology for the two genera. This preliminary results suggest that these aperture ontogeny profiles could aid the taxonomist in decision-making for grouping taxa based on homologous characters.

### Quantitative comparison between different shell forms

Fig 5 shows dendrograms resulting from a permutation distribution clustering analysis of the eight shells in terms of their aperture ontogeny profiles. Fig 5A shows the hierarchical clustering of the eight shells based on all four aperture ontogeny profiles. From this dendrogram, the composite *Opisthostoma vermiculum* is completely separate from the other shells. The remaining seven shells are clustered into two groups. One consists of the more regularly coiled shells, namely, *Plectostoma christae* and its two transformed shells, and *Plectostoma crassipupa*; the other group consists of the shells that deviate from regular coiling, namely *Plectostoma laidlawi* and its transformed shell, and *Opisthostoma vermiculum*. Nevertheless, there were high dissimilarities between shells within each group as revealed by the long branch lengths in Fig 5A, except for the two *Plectostoma laidlawi* shells (Table 1). The aperture ontogeny profiles for the *Plectostoma laidlawi* shell and its reduced version are almost the same. The high dissimilarity among the other six shells can be explained when each of the variables in the aperture ontogeny profile is analysed separately as shown in Fig 5B.

**Table 1:** Dissimilarity matrix of aperture ontogeny profiles of eight shells obtained from Permutation Distribution Clustering.

Shell	(1)	(2)	(3)	(4)	(5)	(6)	(7)	(8)
(1) *Plectostoma laidlawi*	0,00							
(2) *Plectostoma crassipupa*	2,44	0,00						
(3) *Plectostoma christae*	2,65	2,83	0,00					
(4) *Opisthostoma vermiculum*	2,63	2,56	2,59	0,00				
(5) half-sized *Plectostoma laidlawi*	2,69	2,80	0,09	2,55	0,00			
(6) composite *Opisthostoma vermiculum*	3,12	3,48	3,40	3,39	3,34	0,00		
(7) elongated *Plectostoma christae*	2,09	2,55	3,03	2,79	3,03	3,36	0,00	
(8) depressed *Plectostoma christae*	2,01	2,73	3,16	2,94	3,21	3,84	2,62	0,00

Fig 5B shows the dendrograms of aperture ontogeny profiles for each of the four variables. All four dendrograms have a different topology than the one in Fig 5A. Among the variables, the aperture ontogeny profile of the curvature has the smallest discrepancies among shells. The two *Plectostoma laidlawi* shells are the only pair that clusters together in all the dendrograms of Fig 5A and 5B because they are identical in every aspect of aperture ontogeny profile except torsion. Hence, the independent analysis of aperture ontogeny profile variables corresponds well to the overall analysis of aperture ontogeny profiles. The analysis of PDC is based on the standardised ontogeny profiles and their trends. Thus, it is useful for the comparative analysis of shell shape, but not shell size. Nevertheless, the size comparison between shells is rather straightforward.

In this study, we quantified the shell size as shell volume, which can be estimated easily from retopologised 3D shell models (Fig 4). This quantification of shell size in terms of volume is more meaningful from the functional and developmental point of view because a snail should grow a shell in which its entire soft body can fit when the snail withdraws into the shell. We can then compare the form between shells when the dendrograms are interpreted together with shell size (volume) data. For example, the *Plectostoma laidlawi* shell has the same shape as, but is eight times larger than, the resized *Plectostoma laidlawi*.

In addition to the construction of morphospace, the dissimilarity matrix can be used in phylogenetic signal tests [107]. Furthermore, it can also be analysed together with other distance matrices, such as for geographical or ecological distance, to improve our understanding of the evolutionary biology of shell forms.

### Conclusions, Limitations and Future Directions

We demonstrated an alternative workflow for data acquisition, exploration and quantitative analysis of shell form. This method has several advantages: (1) robustness—this method can be used to compare any shell form: The same aperture profiles can be obtained from any form of shell. Then, these profiles from different shells and/or different studies can be analysed together. These parameters can be obtained from the aperture as long as the shell grows accretionarily at the aperture; (2) scalability and reproducibility—the data obtained from different studies and different gastropod taxa can be integrated: Aperture ontogeny profiles were obtained from the aperture outlines. This is a trait that exists in every gastropod shell. We believe that the aperture outline that is obtained by multiple experienced malacologists, on different shells, would be highly similar; (3) versatility—outputs from this method are comply with data standard that is required in taxonomy (e.g., functional morphology, theoretical modelling, and evolutionary studies: the raw 3D shell mesh models can be used for visualisation of shells in taxonomic research (e.g. [76]), coordinates data of the vertices can be used for theoretical modelling (e.g. [2]), aperture ontogeny profiles can be used for shell functional studies [108], and dissimilarity matrix between shell forms can analysed with phylogenetic distance matrix.

Yet, our method has its limitations. Firstly, our retopology procedures rely on a 3D shell model that requires CT-scan technology. In fact, although a CT-scan 3D shell model can certainly facilitate the retopology process of a shell, it is not indispensable. The key of the retopology processes is to digitise the aperture along the shell ontogeny, and thus a shell can be retopologised fully in Blender with a good understanding of the aperture ontogeny profiles by studying the real specimens even without a reference shell model. Secondly, the retopology procedure which is essential for our data acquisition is more time-consuming than traditional and geometric morphometric where data can be obtained from an image taken from a shell. Thirdly, our method is effective in the analysis of overall shell form, but not of the shell ornamentation.

In the future, our method can be improved to accommodate the shell ornamentation analysis. Parts of our method (i.e. procedures 1–6) can be used to obtain shell ornamentation data, such as radial ribs (*i*.*e*., commarginal ribs), but these data cannot be analysed with our qualitative and quantitative approaches that focus on longitudinal growth (i.e. procedures 7–8). Finally, we hope this shell form quantification method will simulate more collaboration within malacologists that work in different research fields, and between empirical and theoretical morphologists.

## Supporting Information

S1 Protocol A step-by-step manual. (PDF)

S1 Movie Video tutorial for procedure 3 and 4. (MP4)

S1 Text A python script for procedures 5 and 6 –Aperture form and growth trajectory analysis on retopologised 3D shell mesh in Blender. (TXT)

S2 Text A python script to convert normalised elliptical Fourier coefficients to polygon mesh in Blender. (TXT)

S3 Text An R script for data analysis as described in procedures 7 and 8. (R)

S1 Dataset A Blender file consisting of raw data of 8 shells of procedures 1–4. (BLEND)

S2 Dataset PLY ASCII mesh 3D model of *Plectostoma laidlawi* Sykes 1902. (PLY)

S3 Dataset PLY ASCII mesh 3D model of *Plectostoma crassipupa* van Benthem Jutting, 1952. (PLY)

S4 Dataset PLY ASCII mesh 3D model of *Plectostoma christae* Maassen 2001. (PLY)

S5 Dataset PLY ASCII mesh 3D model of *Opisthostoma vermiculum* Clements and Vermeulen, 2008. (PLY)

S6 Dataset PLY ASCII mesh 3D model of *Plectostoma laidlawi* that was reduced in size by one-half and with slight modification of the last aperture size. (PLY)

S7 Dataset PLY ASCII mesh 3D model of *Plectostoma christae* that was reshaped into an elongated form by reducing the model size (linear dimension) by one-half along the x and y axes, and by doubling the size along the z axis. (PLY)

S8 Dataset PLY ASCII mesh 3D model of *Plectostoma christae* that was reshaped into a depressed form by doubling the model size along the x and y axes, and by reducing the size by one-half along the z axis. (PLY)

S9 Dataset PLY ASCII mesh 3D model of *Opisthostoma vermiculum* that consists of one *Opisthostoma vermiculum* original 3D model of which the aperture was connected to a second enlarged *Opisthostoma vermiculum*. (PLY)
