# Supplementary material for: A Method for Quantifying, Visualising, and Analysing Gastropod Shell Form
Source: PLoS One. 2016 Jun 9;11(6):e0157069. doi: 10.1371/journal.pone.0157069 (PMC4900530; doi:10.1371/journal.pone.0157069)
Supplement: S1 Protocol — (PDF) [file pone.0157069.s001.pdf]

MANUAL

A method for quantifying, visualising, and analysing gastropod shell form

This manual provides detailed step-by-step instructions that guide you through the procedures used in this method.

1. **Obtaining digital 3D models from actual shells**
2. **Pre-processing digital shell models**
3. **Creating reference: Tracing aperture outlines and ontogeny axis from shell models**
4. **Retopologising aperture outlines from the reference and generating retopologised shell models**
5. **Quantifying aperture growth trajectory**
6. **Quantifying aperture form**
7. **Visualising aperture form and trajectory changes along the shell ontogeny**
8. **Quantitative comparison between shell forms**

This manual describes the procedures from Step 3 until Step 8. For Steps 1 and 2, please refer to the manufacturer's manual of the CT-scanner. For Steps 7 and 8, users have to have basic knowledge of R packages. The complete R script for Steps 7 and 8 is available in Supplementary Materials File 5. In addition, the user can also refer to the Video tutorial: Supplementary Materials File 2.

This manual is not intended to help users to familiarise themselves to the R packages, Python programming language, and Blender software as there are already comprehensive texts and video tutorials available elsewhere, for example:

- R
  - https://youtu.be/WJDrYUqNrHg?list=PL6FB1B1E94F292136
- Python
  - https://youtu.be/4Mf0h3HphEA?list=PLEA1FEF17E1E5C0DA
- Blender
  - https://www.youtube.com/playlist?list=PL03BAC38A5E823407

Before starting
It is important to set Blender User Interface and MOUSE. For MOUSE setting, go to File > Blender User Preferences > Tick “Emulate 3 Button Mouse”.

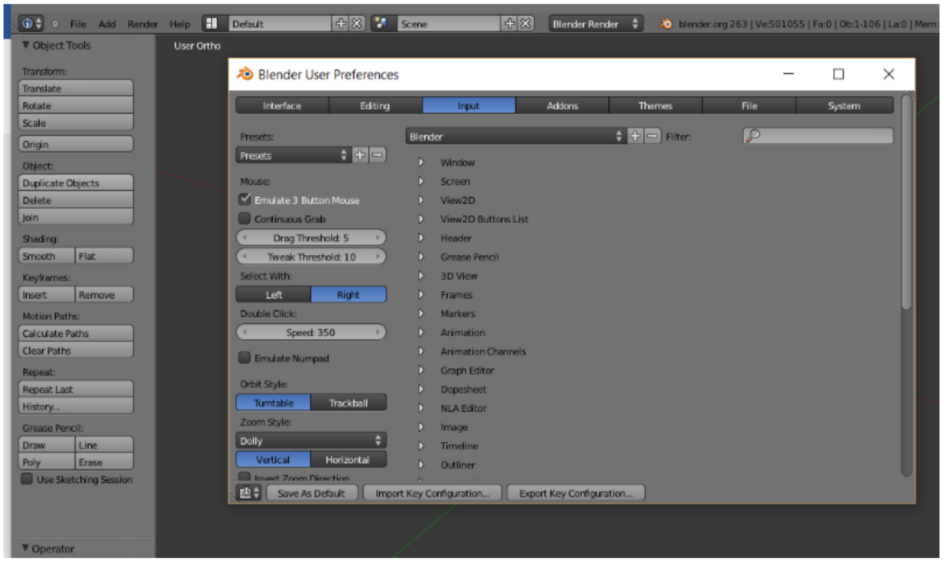

Blender Interface needs to be set so that the following four **Windows** are shown: **(1)** Python Console, **(2)** Outliner, **(3)** 3D View, and **(4)** Properties. In the 3D View and Properties Windows, there are several **Panel**s. There are a total of 20 **Layer**s in the 3D View Window.

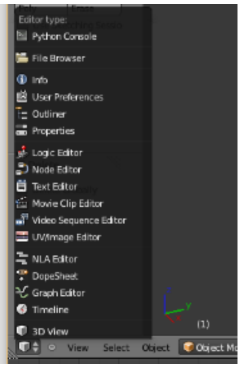

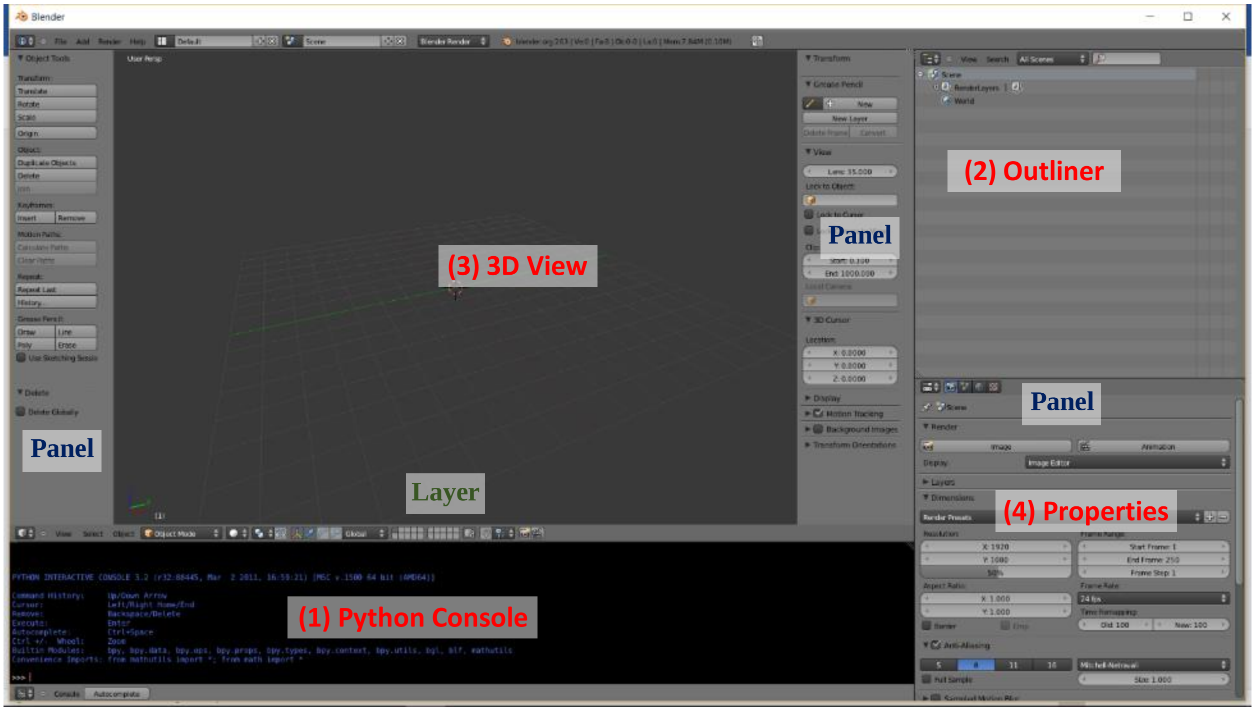

Glossary

Hit once keyboard key:

ENTER
SHIFT
ALT
CTRL
TAB
A
C
D
F
G
M
R
S
X
W

Hold key

HOLD A,
HOLD C,
etc.

Mouse action

RIGHTMOUSE – Click right mouse button
LEFTMOUSE – Click left mouse button
HOLD RIGHTMOUSE/LEFTMOUSE – Hold right mouse button or hold left mouse button
Move MOUSE – move the mouse
Scroll MOUSE WHEEL – Scroll mouse wheel (up or down)
Click – Click on the dropdown menu, on the 3D model, on the panel option etc.
Tick – Tick the radio box or checkbox
Select – Usually use mouse to select 3D models
Adjust – Select and then move the 3D models, control points by using mouse

User activities

Label – Typing the name to label the Grease Pencil drawings, Bezier Curves, NURBS surface circle, etc.
Check – Examine and record the data, condition, etc.

Editing mode in 3D View Window

*Hit TAB to enter edit mode or exit edit mode (enter Object mode)
EDIT mode – To edit 3D model
OBJECT mode – To manipulate 3D model

Annotation used in the descriptions of STEP 3, STEP 4, STEP 5, and STEP 6.

(   ) – Mouse or Keyboard actions
“   ” – Panel name or Option name
> **-** Hit one key after another.
+ **-** Press more than one key at once.

STEP 3 Creating reference: Tracing aperture outlines and ontogeny axis from shell models (Video tutorial from 00:30 to 21:40)

1. Import 3D model

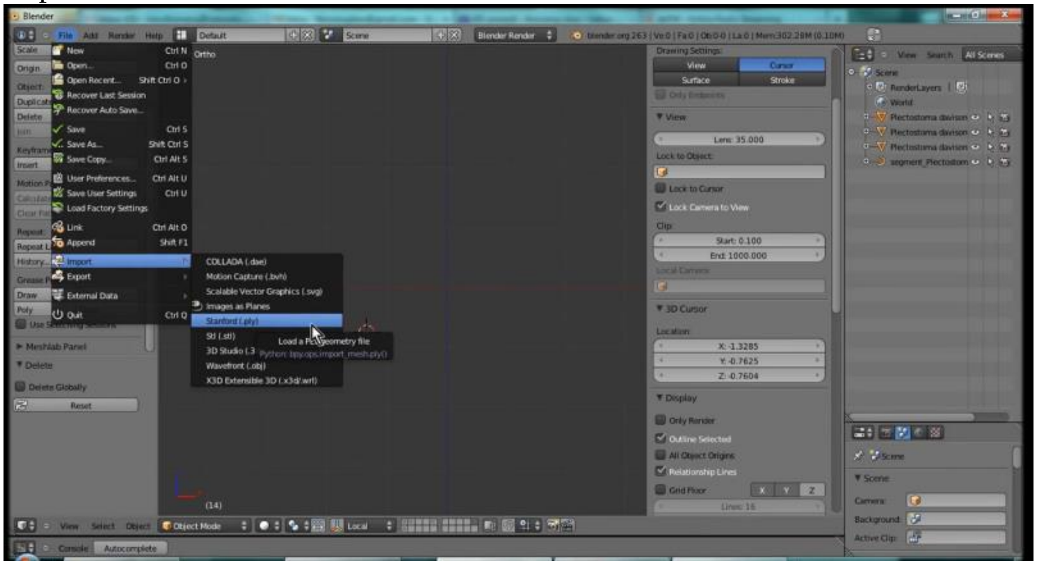

2. Select (RIGHTMOUSE) and scale up model (S > 1000 > ENTER). Then apply the scaling (CTRL + A > S).
   Note: The model in this study is very small with dimension about 0.002 blender unit. Hence, we enlarged the model 1000 X, so that 1 blender unit of the model is equal to 1 mm.

3. Manipulation of the Scene in the 3D View window: For orientation: ALT + HOLD RIGHTMOUSE > Move MOUSE; Zoom in and zoom out – Scroll MOUSE WHEEL.

4. In the 3D View window, open Grease Pencil Panel. Click “New” > then Click “New Layer”.

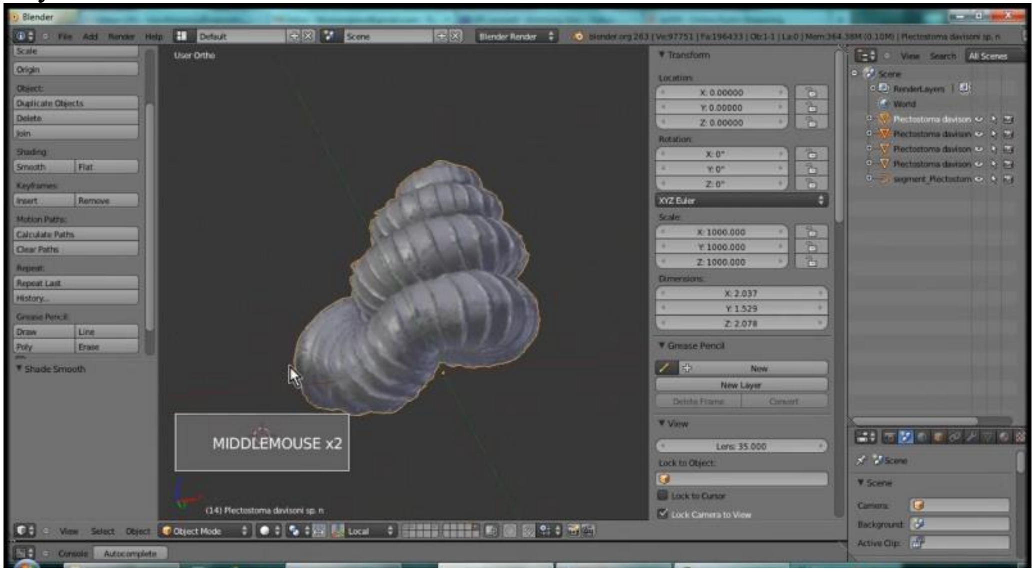

5. Go the “Drawing Settings”, then Click “Surface”

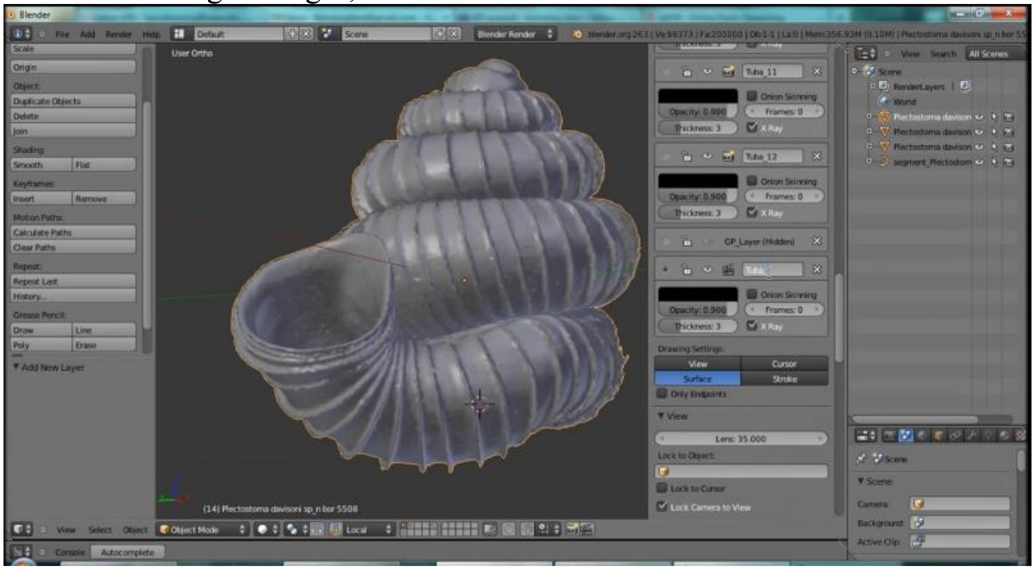

6. Select the colour of the Grease Pencil, and Label the aperture drawing.

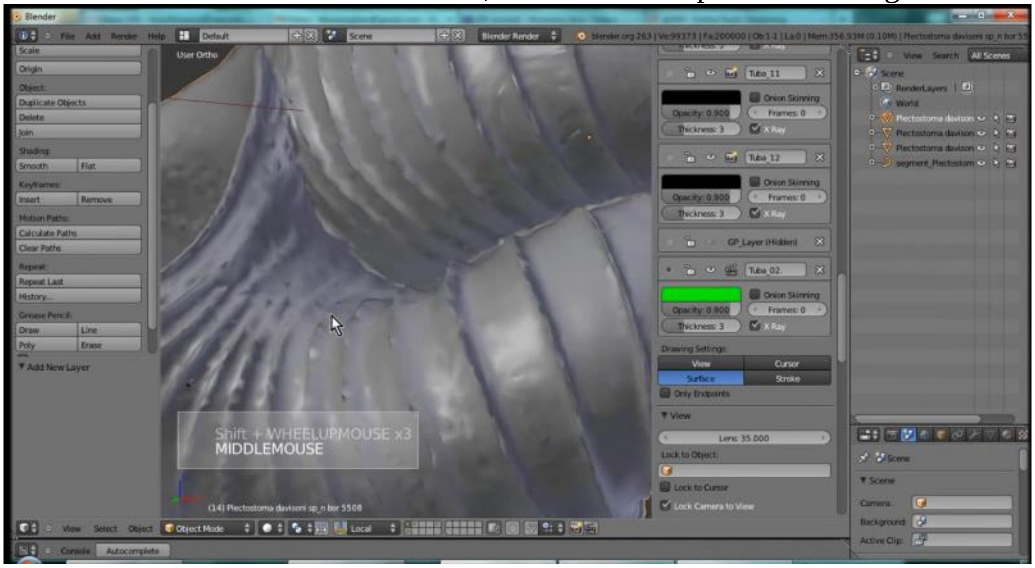

7. Tracing aperture outline of the outer shell surface: CTRL + D > HOLD LEFTMOUSE > Move MOUSE. For each new aperture outline, Click “New Layer” (see Step 4), and repeat this step to trace all the aperture outlines.

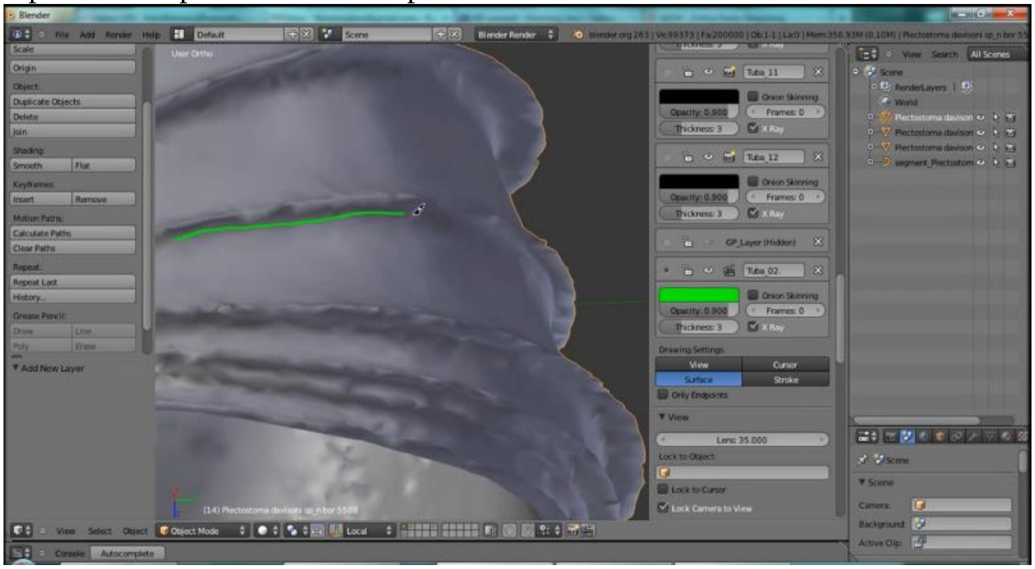

8. Part of the 3D shell model might need to be clipped away to draw the line on the inner shell.

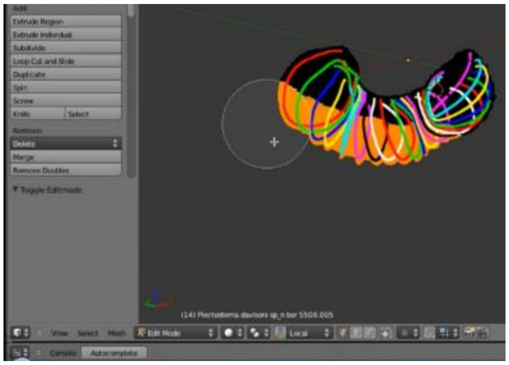

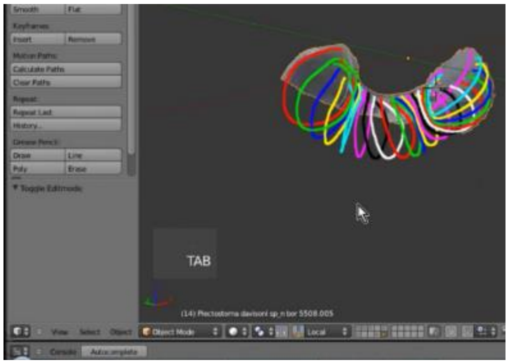

9. To clip away part of the model, select model (RIGHTMOUSE) > enter edit mode (TAB) > select to part of model (HOLD C + HOLD LEFTMOUSE) > delete the select part (X)
Note: You can check the actual shell of a sub-adult for which its aperture was at the growth stage as the 3D model, to gain some idea about the shape of the aperture outline that is covered by overlapping whorls.

10. Convert the grease pencil tracings to Bezier Curve. In Grease Pencil Panel, Select one aperture grease pencil tracing (Check the radio button), Click “Convert”, Select “Bezier Curve”). Repeat this step for all aperture tracings.

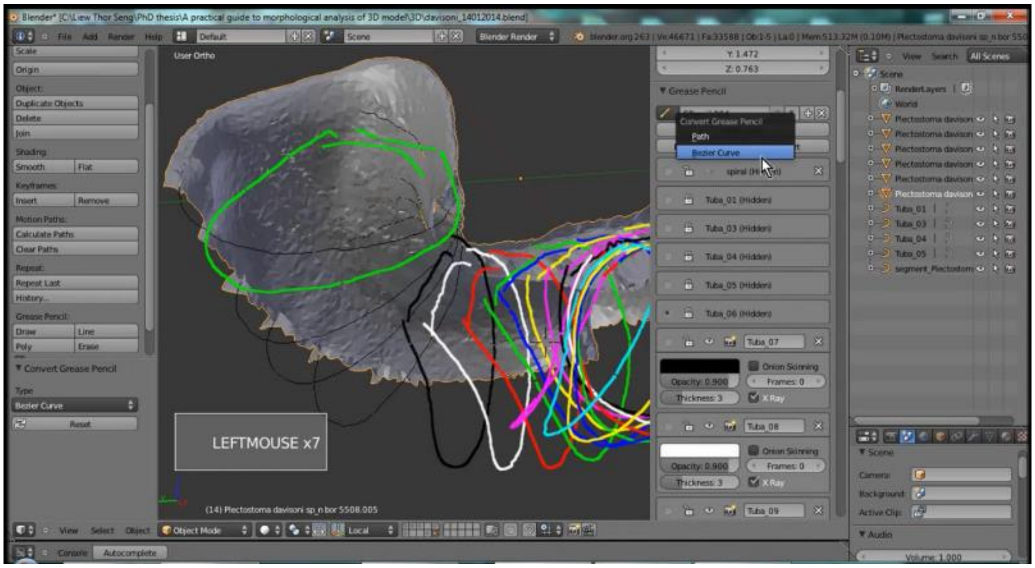

11. Remove the 3D shell model and keep only the Bezier Curve.

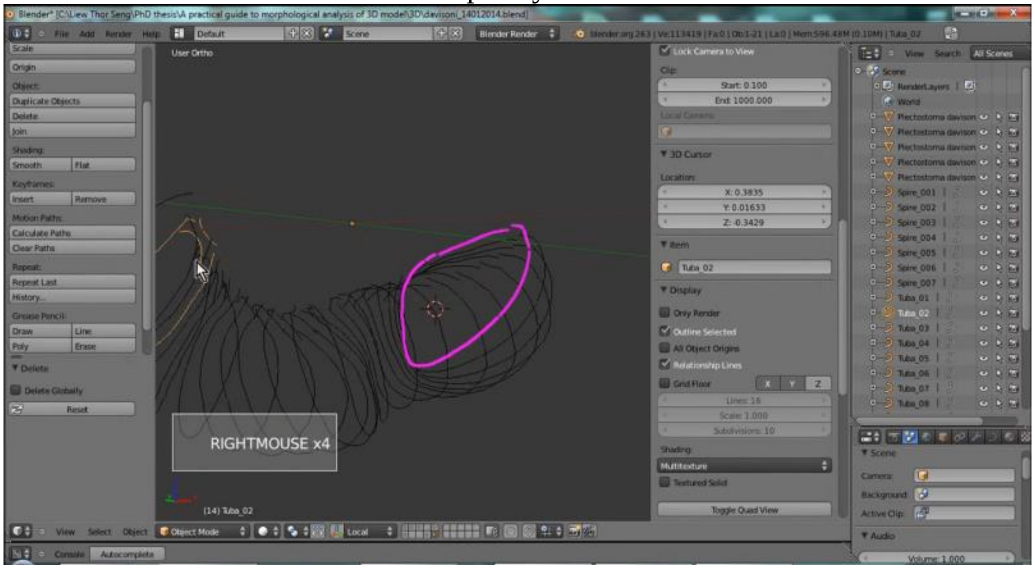

STEP 4 Retopologising aperture outlines from the reference and generating retopologised shell models
(Video tutorial from 21:41 to 55:00)

1. Identify and trace the ontogeny axis from the actual shell, then trace the ontogeny axis by using the Grease Pencil and convert the pen tracing to Bezier Curve (similar to procedure to trace aperture ooutline: step 7 in STEP 3).
   Note: See Liew et al. (2014) for definition of ontogeny axis.
      Thor-Seng Liew, Annebelle CM Kok, Menno Schilthuizen, Severine Urdy. 2014. On growth and form of irregular coiled-shell of a terrestrial snail: Plectostoma concinnum (Fulton, 1901) (Mollusca: Caenogastropoda: Diplommatinidae). PeerJ 2: e383.

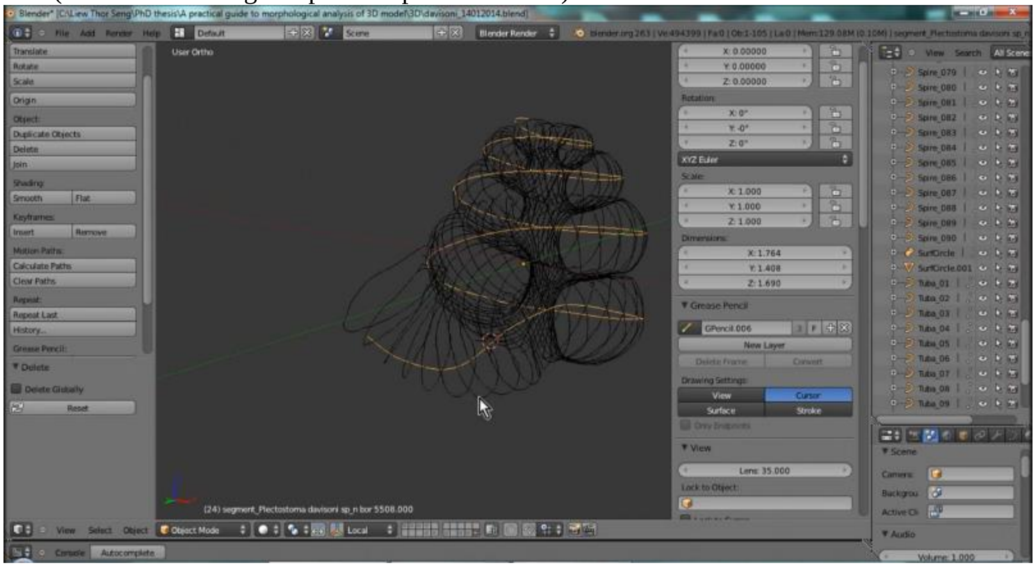

2. Move this ontogeny axis (Bezier Curve) into a new "Layer".

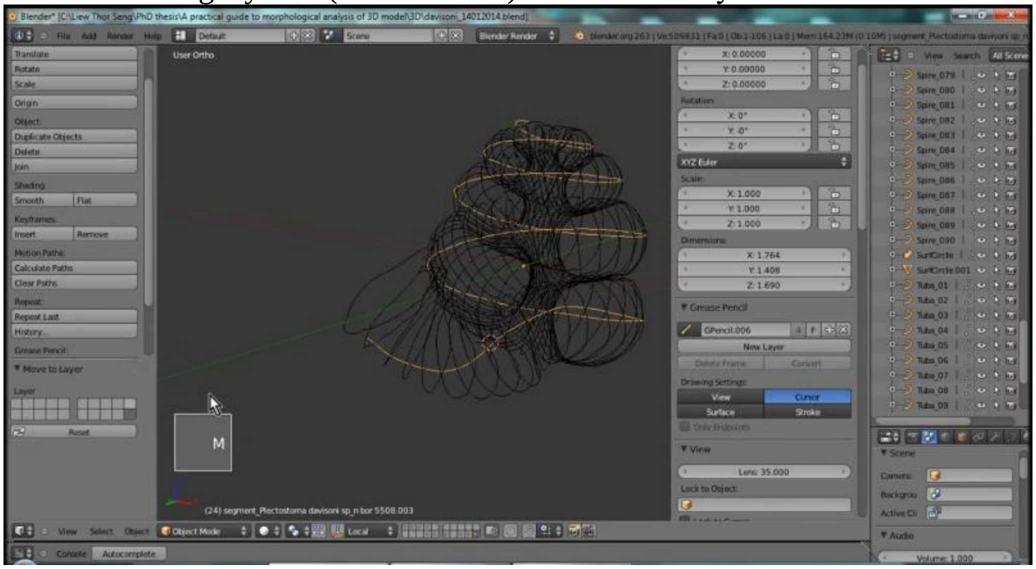

3. Move the first aperture outline (Bezier Curve) into the “Layer” that has the ontogeny axis.
Note: To retopologise the aperture outline one after another.

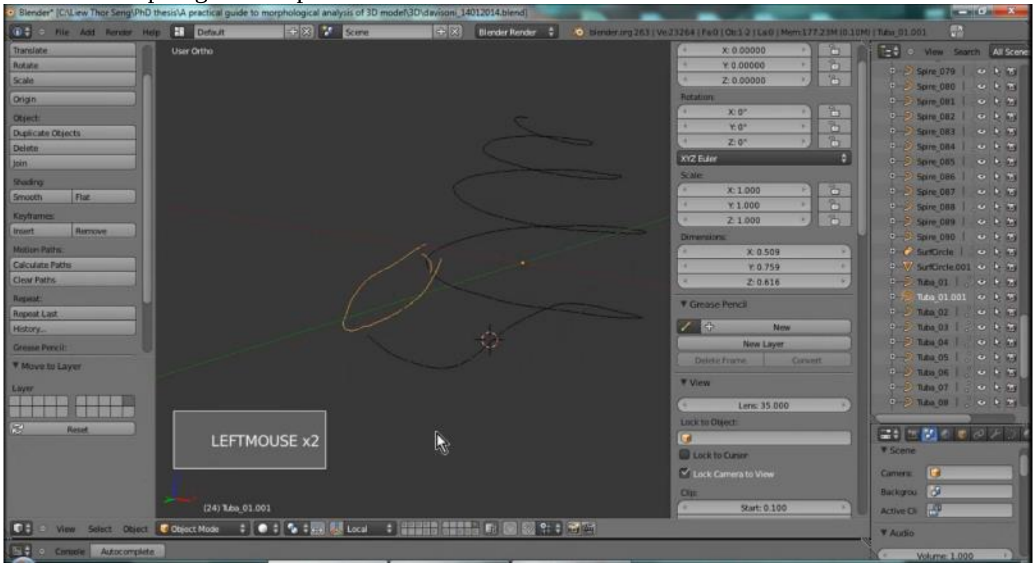

4. Add a new NURBS surface circle into the Layer.

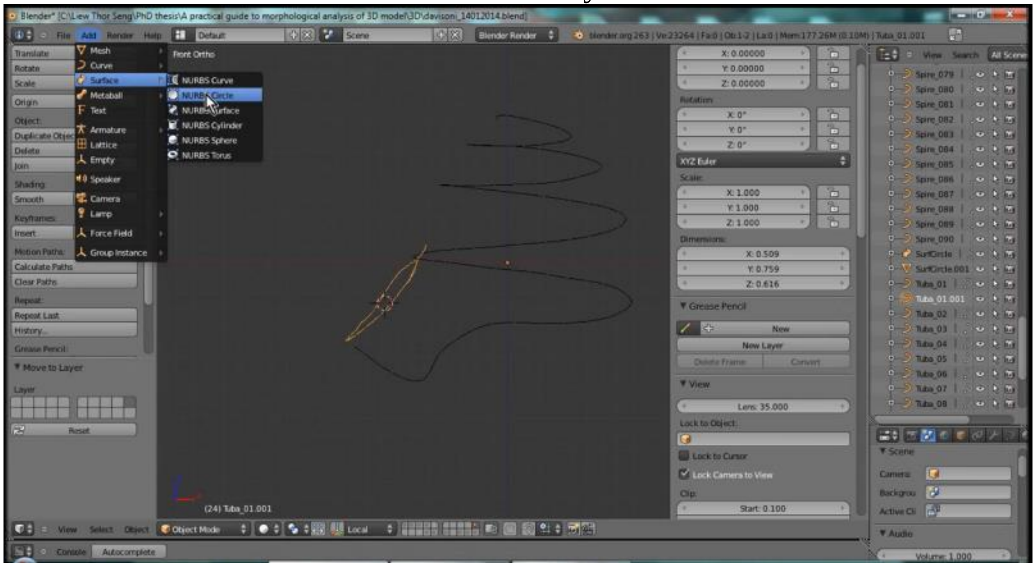

5. Resizing Translating, and orientating the entire surface circle so it at the same orientation as the aperture outline.
   Note: Selecting NURBS surface circle: RIGHTMOUSE; Resizing: S + move MOUSE + LEFTMOUSE; Translating: G + move MOUSE + LEFTMOUSE; Orientating: R + move MOUSE + LEFTMOUSE.

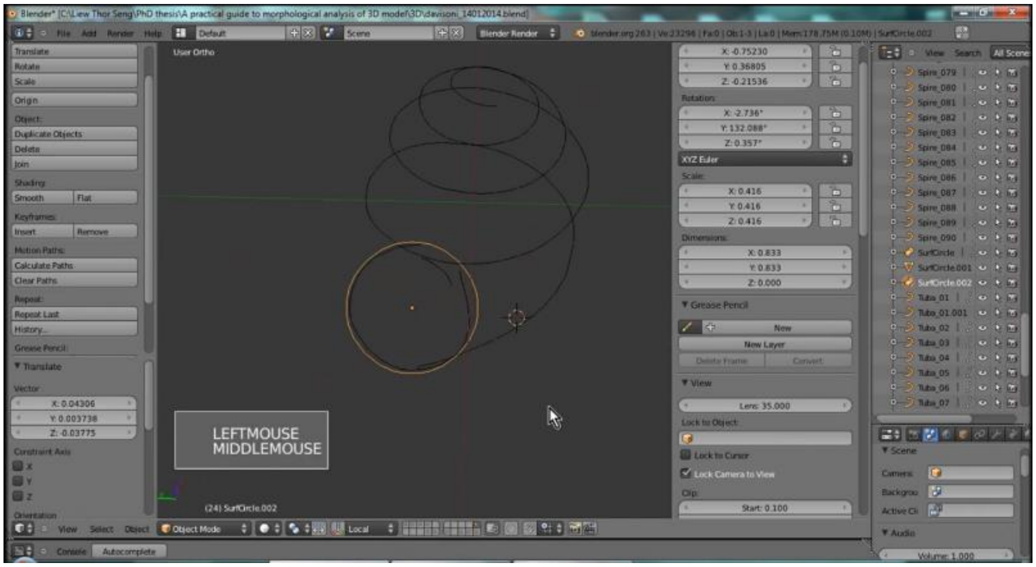

6. Make sure one of the control points in NURBS surface circle is always aligned to the ontogeny axis.
   Note: If the first control point of the first NURBS surface circle was used for the first aperture to align with ontogeny axis, the first point of the following NURBS circle of the following apertures need to be aligned to the ontogeny axis as well.

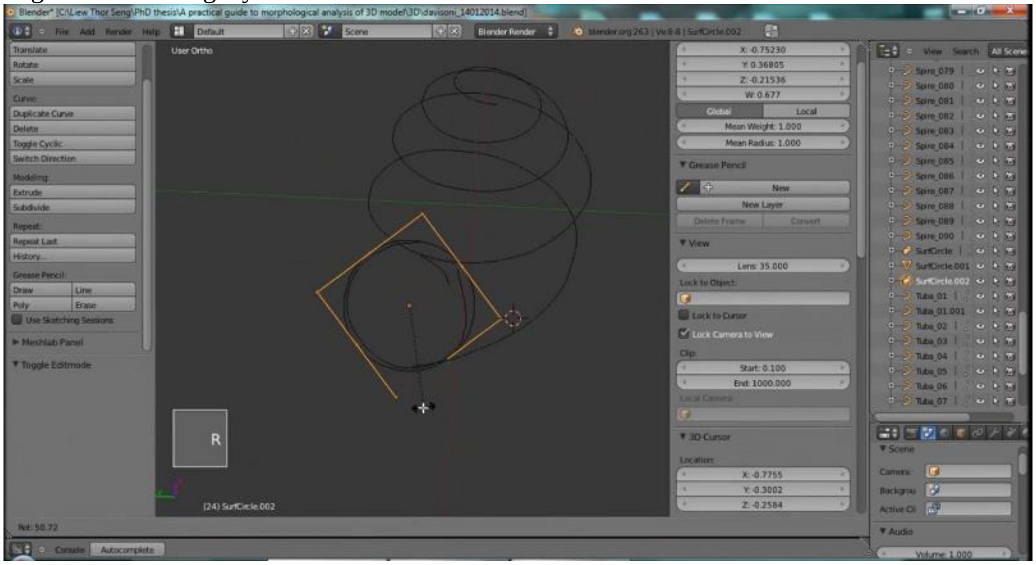

7. Increasing the number of control points in the NURBS surface circle by selecting the circle: RIGHTMOUSE > TAB > W > ENTER
Note: Subdividing the circle until there are enough control points in the circle to control the outline of the circle so that its line aligned completely with the aperture. In most cases, one subdivision that creates 16 control points is enough for apertures that are not too complex.

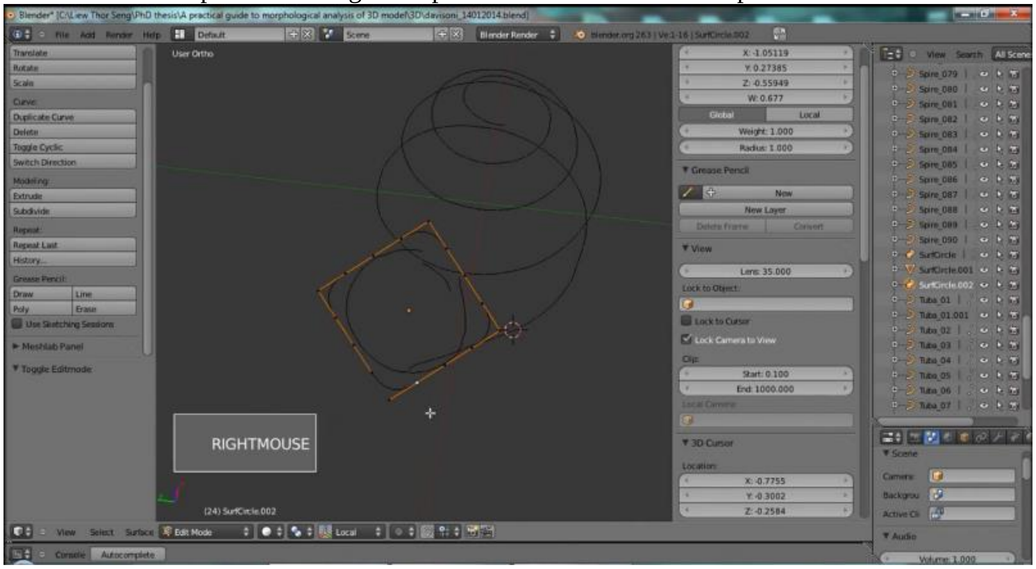

8. Select the same NURBS surface circle, enter EDIT mode (TAB), select each control point in the circle (RIGHTMOUSE), and move each control point (G + move MOUSE) so that the circle outline is aligned to the aperture outline. Make sure one of the control point is aligned to the ontogeny axis (see Step 6).
Note:

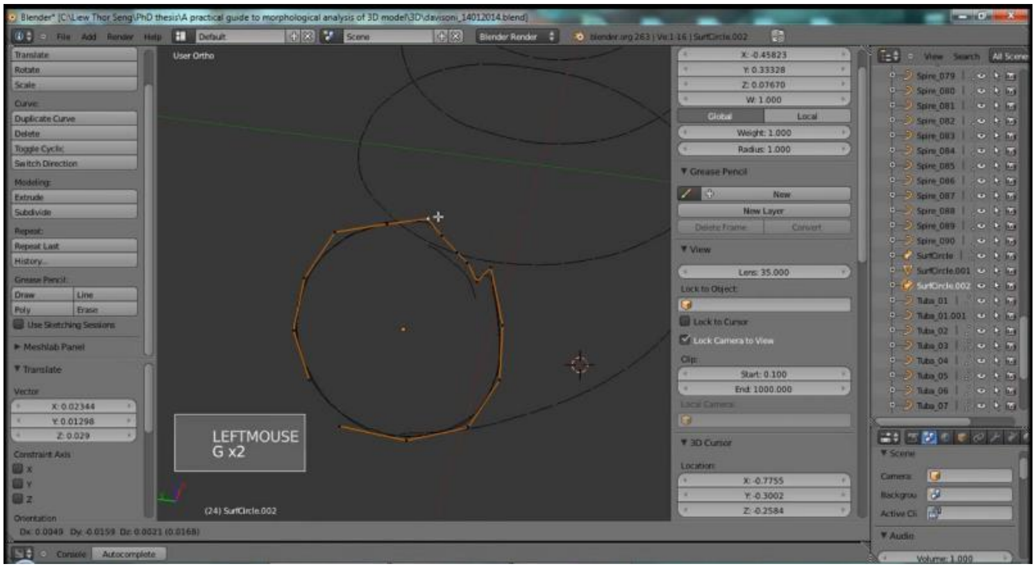

9. Exit EDIT mode (TAB), Check the position of the control point that aligned. Convert NURBS surface circle to MESH. Select mesh object (RIGHTMOUSE) > ALT + C > M. Check the first vertex of the mesh (i.e. number 0) to see if it is aligned to the ontogeny axis.
Note: Select the mesh object (RIGHTMOUSE), then open the Console windows, type the comment: bpy.context.active_object.data.vertices[0].select=True. After that enter the EDIT mode to check the vertices and ontogeny axis.

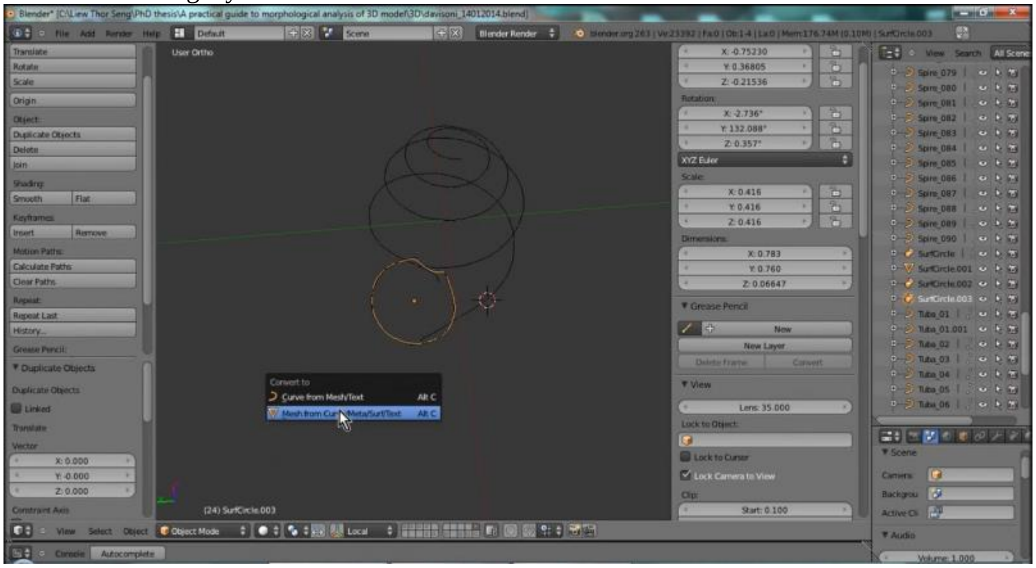

10. If the vertex no. 0 is not aligned to the ontogeny axis, repeat Step 8 and Step 9, until the vertex no. 0 is aligned to the ontogeny axis. Once this is done, remove the Mesh circle: Select the Mesh circle (RIGHTMOUSE) > X.

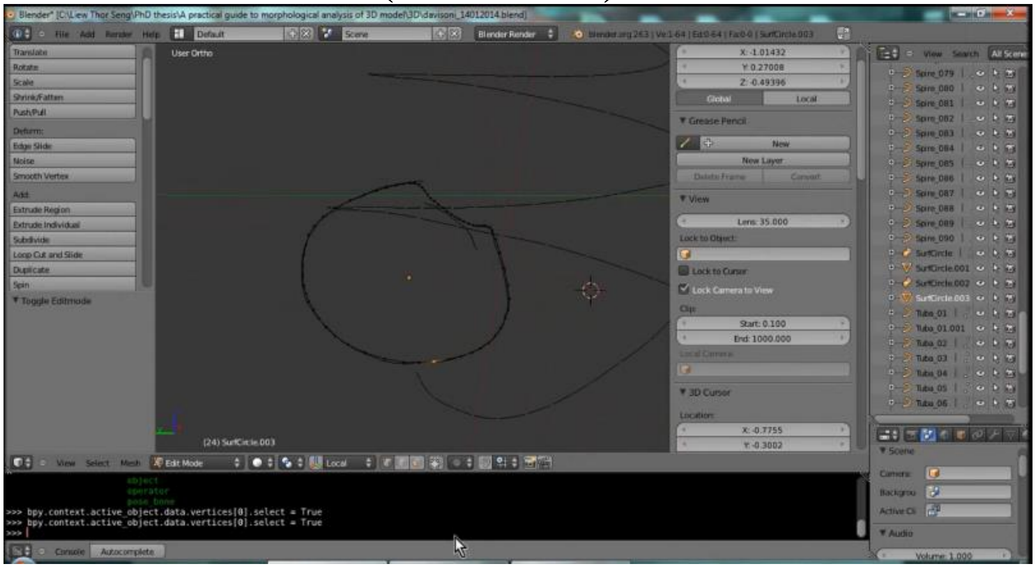

11. Move the second Bezier Curve aperture to the same Layer (see Step 3).

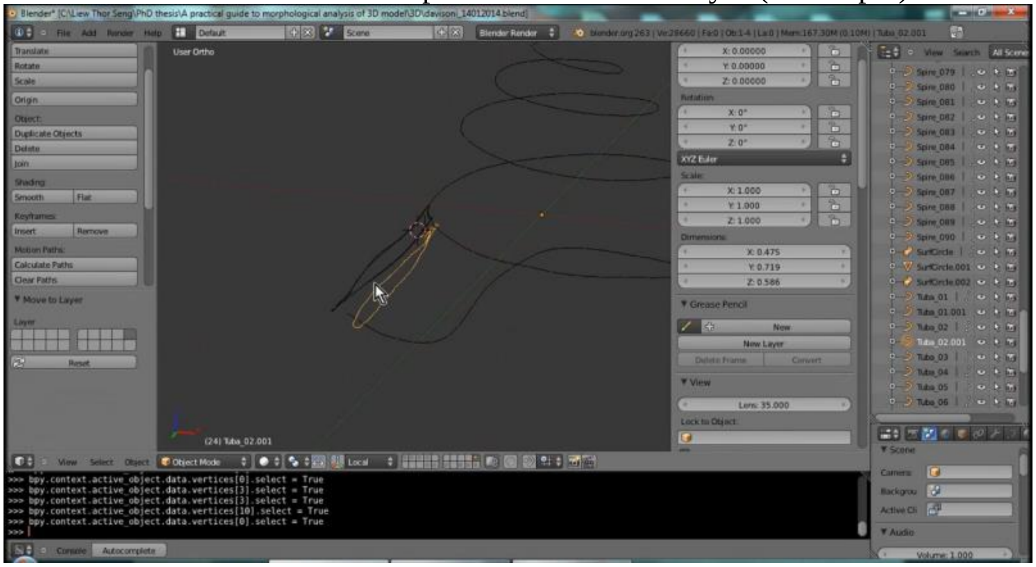

12. Select the first aperture (RIGHTMOUSE) and paste the python script below in Console window and ENTER:

```
shell = bpy.context.active_object
number_of_apertures = len(bpy.context.active_object.data.splines)
number_of_points_for_each_aperture = len(bpy.context.active_object.data.splines[0].points)
bpy.context.scene.objects.active  = shell
shell.select = True
for each_point in list(range(0,number_of_points_for_each_aperture)):
    bpy.context.active_object.data.splines[(number_of_apertures-1)].points[each_point].select = True

bpy.ops.object.editmode_toggle()
```

Note: the second NURBS surface circle is now overlapped with the first aperture, and it is in EDIT Mode.

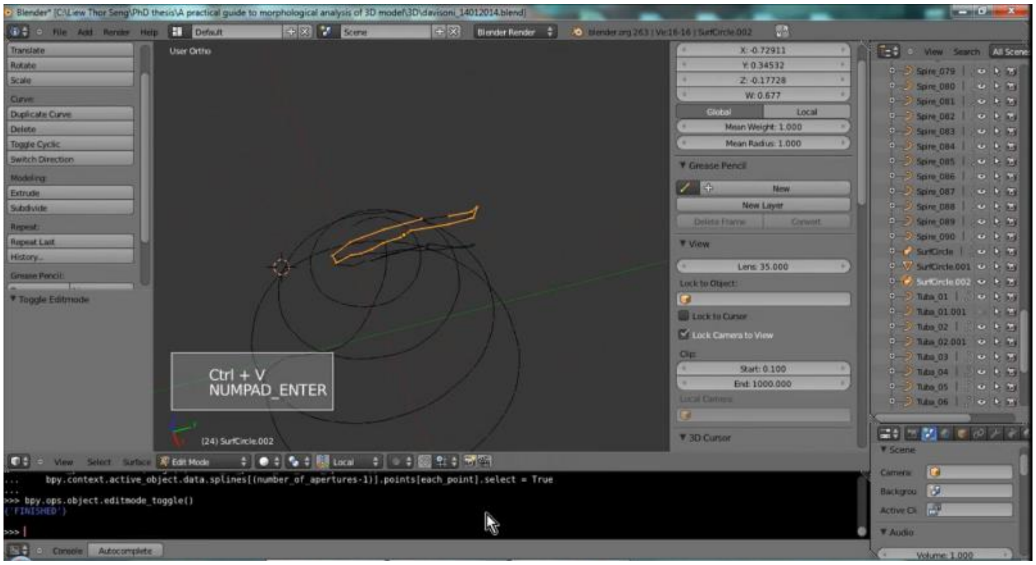

13. Move the entire second NURBS surface circle so that it is aligned with the Bezier Curve for the second aperture (see Step 5).

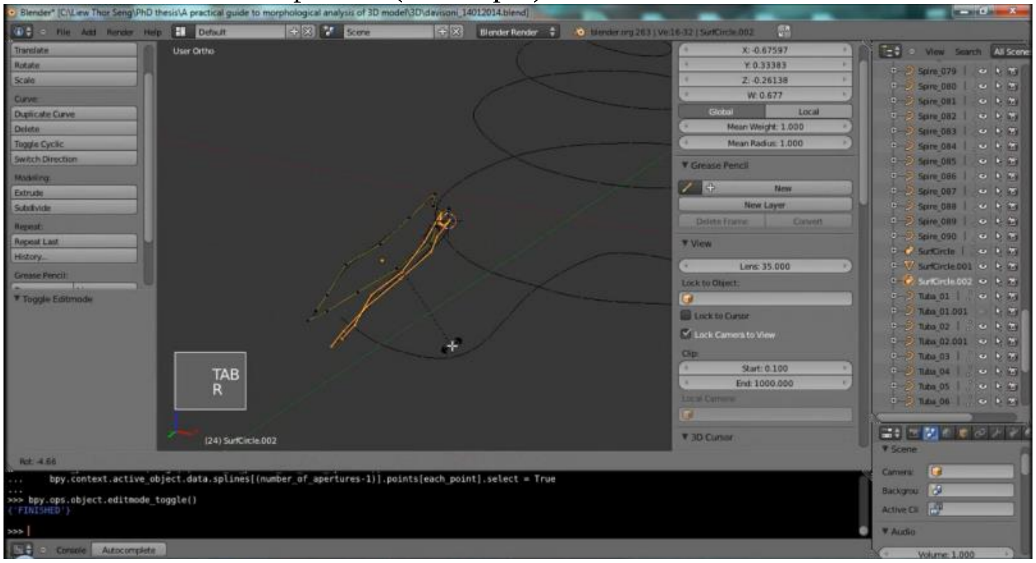

14. Repeat Step 8 for the second NURBS surface circle until it is completely aligned to the second aperture Bezier Curve.

15. Repeat Step 12, Step 13, and Step 14 for the following aperture Bezier Curves in the last shell whorl.

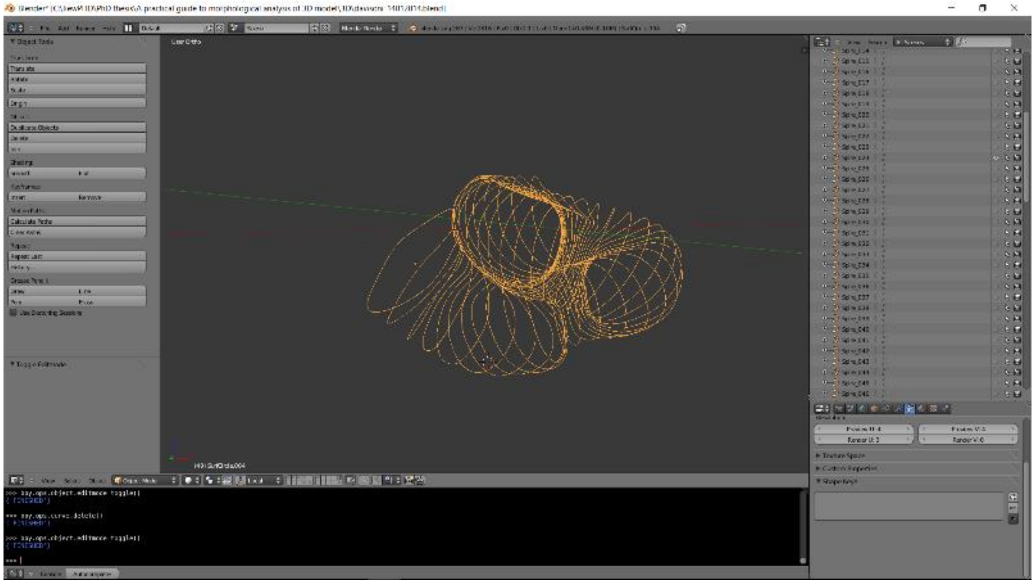

16. After all the aperture's whorl NURBS surface circles in the last shell whorl have been done, in the OBJECT MODE, select and duplicate (RIGHTMOUSE > SHIFT + D > LEFTMOUSE) the NURBS surface circle, which now has several aperture outlines, and move the duplicated NURBS surface circle into a new Layer (M > Select Layer). Note: This step is to create a backup for the NURBS surface circle.

17. Select the NURBS surface circle (RIGHTMOUSE), in the EDIT mode (TAB), create the NURBS surface model (A > F), exit EDIT mode (TAB).
Note: Step 17 and 18 is to fine tune the respective aperture in NURBS surface circle so that the NURBS surface (i.e. shell whorl) of the preceding whorl will not intercalate with the later whorl at the whorl overlapping part.

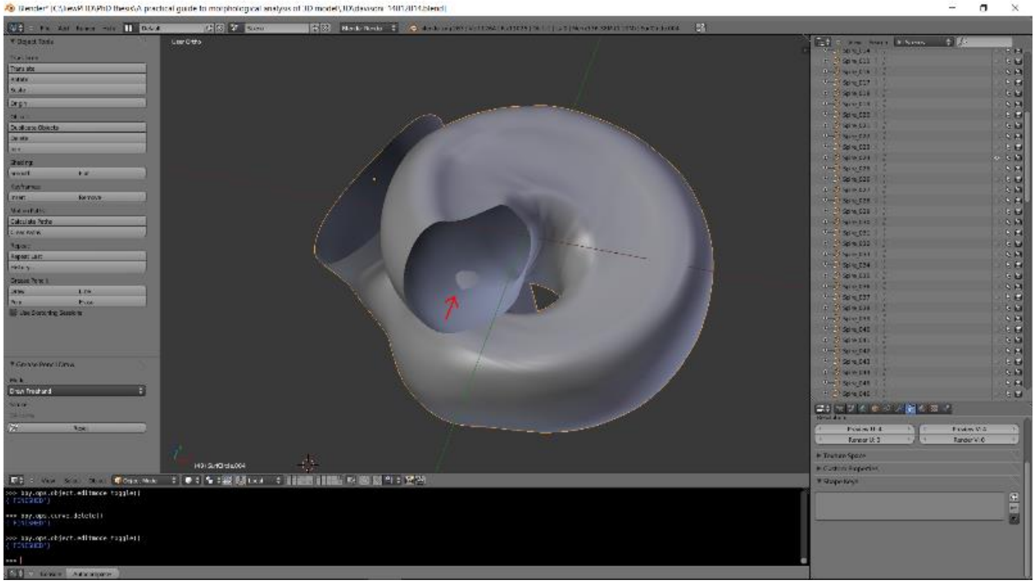

18. If the intercalation between NURBS surface occurs, adjust the control point of the apertures where the surface was interpolated by referring to the Bezier Curve of the particular apertures. Select both of the Layers with NURBS surface model and the original NURBS surface circle (SHIFT + LEFTMOUSE). Select only the NURBS surface circle (RIGHTMOUSE), and in the EDIT mode (TAB), adjust the control points.

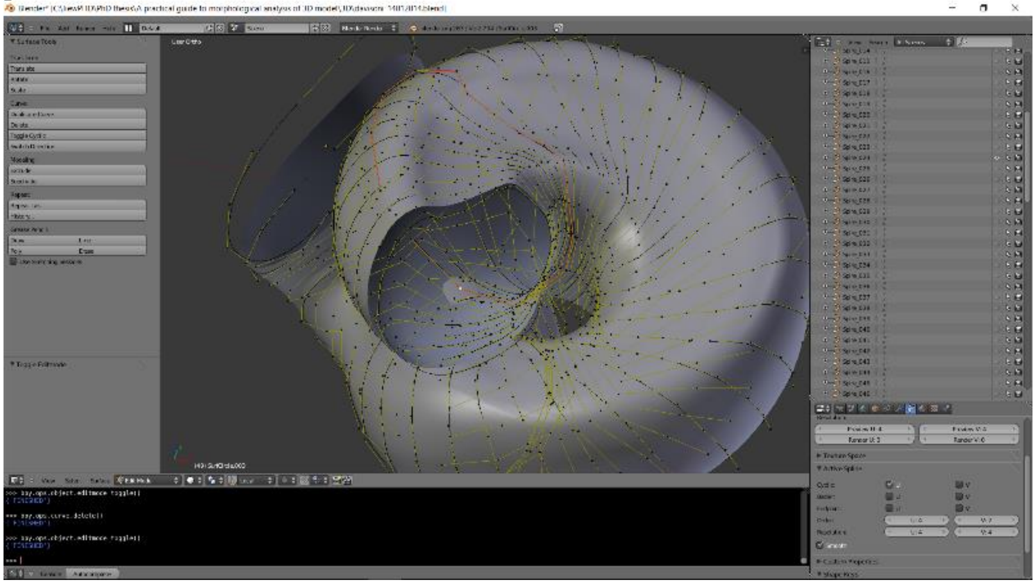

19. Repeat Step 15 for the following 2 – 5 apertures. Then, repeat Step 16 – Step 18.

20. Repeat Step 19 until NURBS surface circles were created from all apertures and a NURBS surface shell model has been created.

21. Select the NURBS surface model (RIGHTMOUSE), enter EDIT mode (TAB), go to Properties Window> “Active Spline” > Tick “Endpoint”.
Note: to extend the surface toward the end of the first and the last NURBS surface circles (i.e. apertures).

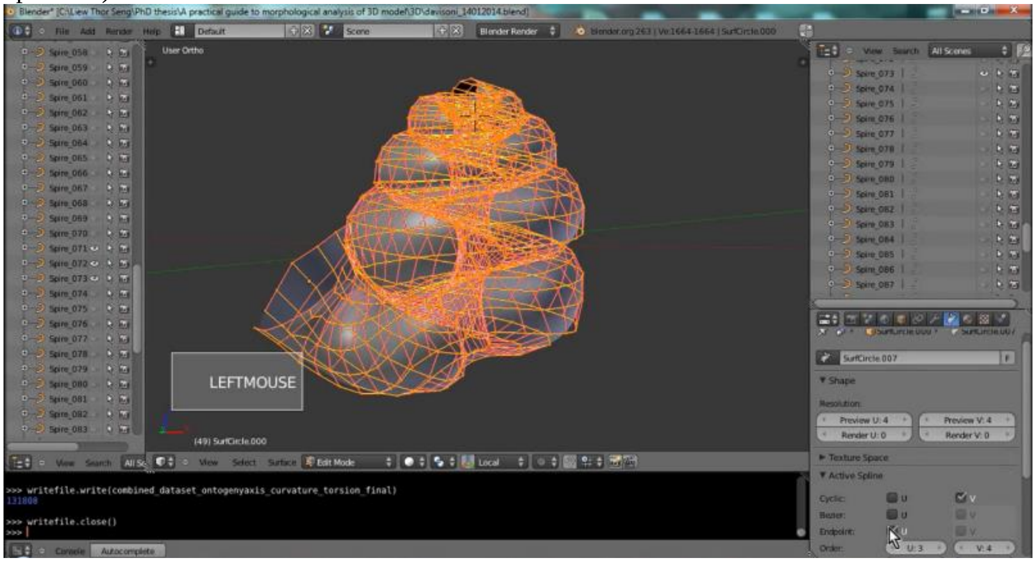

22. In the same Properties Window, increase the resolution of the model: “Active Spline” > Resolution, double the U value.
Note: This step is to increase the resolution of the NURBS surface model so that the MESH model (Step 23) converted from this NURBS surface model will have the highest resolution in terms of the total number of apertures and the number of vertices in each aperture outline.

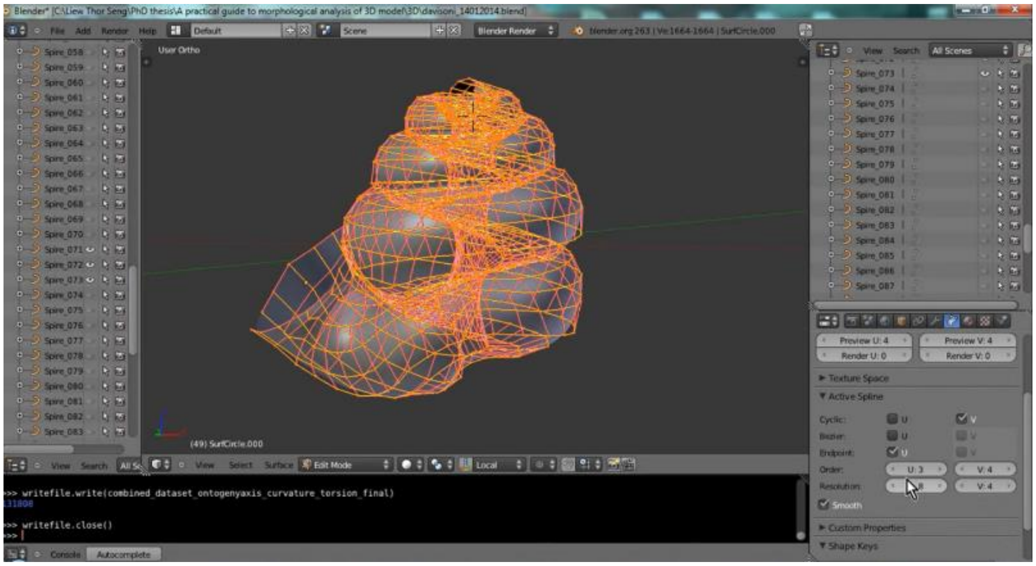

23. In the OBJECT mode, convert the NURBS surface model to MESH model: ALT+ C > M.
Note: This MESH model will be used for quantification of the aperture growth trajectory and aperture form.

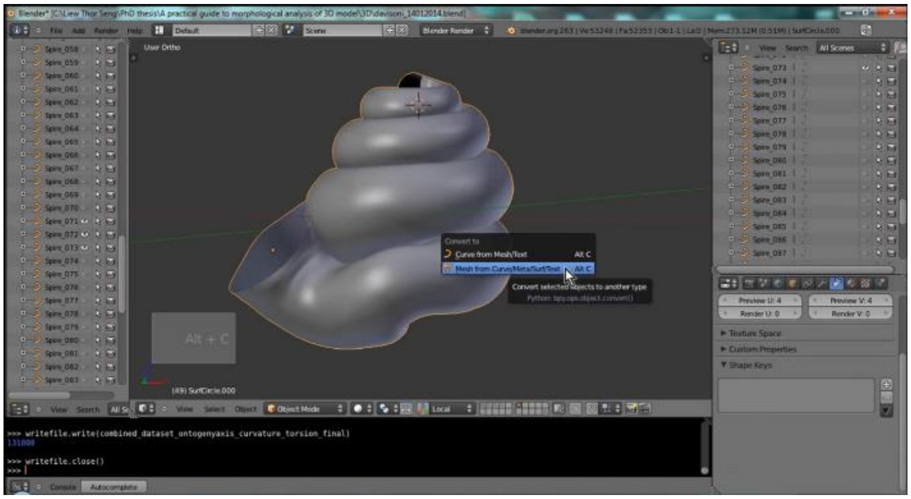

24. Check the number of the vertices (points) for the first aperture.
Note: all the apertures have the same number of vertices.

STEP 5 and 6: Quantifying aperture growth trajectory and aperture form

1. Create a file directory in the computer where the analysis output files will be kept.

2. Open the Python script in Notepad (Supplementary Material File 3)

3. Set the number of the vertices for aperture in the script (See Step 24 in **STEP 4**).

```
len(bpy.context.active_object.data.vertices)/64
```

4. Obtain the total number of the apertures in the MESH model. Select the MESH model (RIGHTMOUSE) and paste the Script (see Step 3) into the Python Console Window.

5. In the same Python script (Step 2), set the number of aperture vertices and parameter **q** for curvature and torsion analysis.
   Note: To minimise the convergence issue when estimating curvature and torsion value, the **q** value needs to be set around 1/10 of the value of the total number of apertures (Step 4).

```
################################################################################################
#                                                                                              #
# Defining the number of points of each aperture and total number of point along ontogeny axis  #
#                                                                                              #
# * Parameter: number_of_points_for_each_aperture                                              #
#                                                                                              #
################################################################################################

number_of_points_for_each_aperture = 64
Total_number_of_points_of_3Dmodel = len(bpy.context.active_object.data.vertices)
Total_number_of_points_of_3Dmodel
number_of_points_for_ontogeny_axis = int(Total_number_of_points_of_3Dmodel/number_of_points_for_each_ap
number_of_points_for_ontogeny_axis
list_of_ontogeny_axis_points = []
First_point_of_aperture = 0
```

```
########################################
#                                      #
# Algorithm for curvature and torsion  #
#                                      #
# * parameter: q                       #
#                                      #
########################################
q=70
a1=a2=a3=a4=a5=a6=0
bx1=bx2=bx3=by1=by2=by3=bz1=bz2=bz3=0
l_list=[]
l_list_center =[]
s_list=[]
wi_list=[]
#list_of_curvature = []
#list_of_torsion = []
```

6. Set the file directory in the Python script.

```
##Parameters(newfolderpath)
fp=bpy.data.filepath
filepath=os.path.basename(fp)
Blender_file_name=filepath[:-6]
newfolderpath="c:/E_19072009/abc/"
if not os.path.isdir(newfolderpath):
    os.makedirs(newfolderpath)

outputfile1= newfolderpath+" "+Blender_file_name+ " " + name +" perimeter"+".csv"
outputfile2= newfolderpath+" "+Blender_file_name+ " " + name +" EFA"+".csv"
outputfile3= newfolderpath+" "+Blender_file_name+ " " + name +" NEFA"+".csv"
writefile=open(outputfile1, 'w')
```

7. Select the MESH model (RIGHTMOUSE), copy the entire Python script and paste it into the Python Console Window. Hit ENTER to execute the Python script.

8. Ontogeny profiles, namely, aperture size, aperture shape, curvature, and torsion of the ontogeny axis will be saved as CSV file in the select File directory.

9. The ontogeny profile can be analysed in R.

STEP 7: Visualising aperture form and trajectory changes along the shell ontogeny

1. Open R script in R.

2. There are five parts of the R script:

```
# line 12   - A) raw data obtained from procedure 1 - 6.
# line 155  - B) Procedure 6: Principal component analysis on EFA coeefficient.
# line 177  - C) Preparing data for procedure 7 and 8.
# line 212  - D) Procedure 7: scatter plots.
# line 633 - E) Procedure 8: cluster analysis.
```

3. Part A of the script is for data preparation. Change the file directory for each of the CSV files for aperture ontogeny profile: ***NEFA.csv, ***perimeter.csv, and ***q##.csv, of each shell.

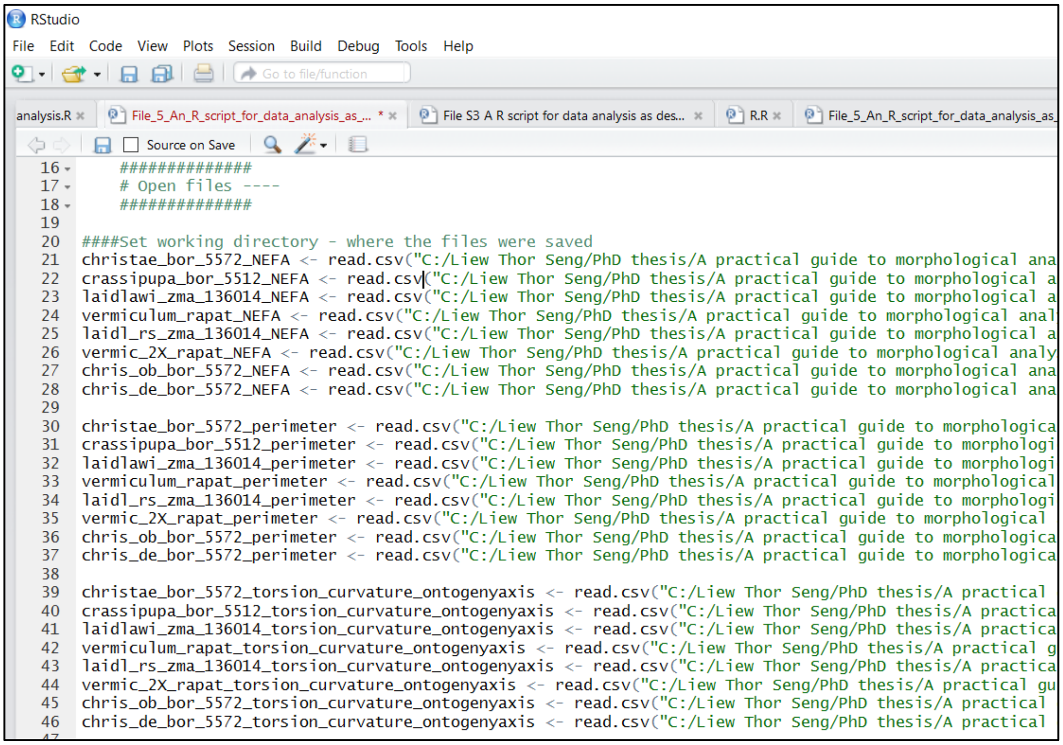

4. Insert shell name (e.g. species name) for the data of the respective shell and the number of apertures (4 in **STEP 5 & 6**). This step is to combine the datasets from different shells.

```
label <- rep('christae', 760)
```

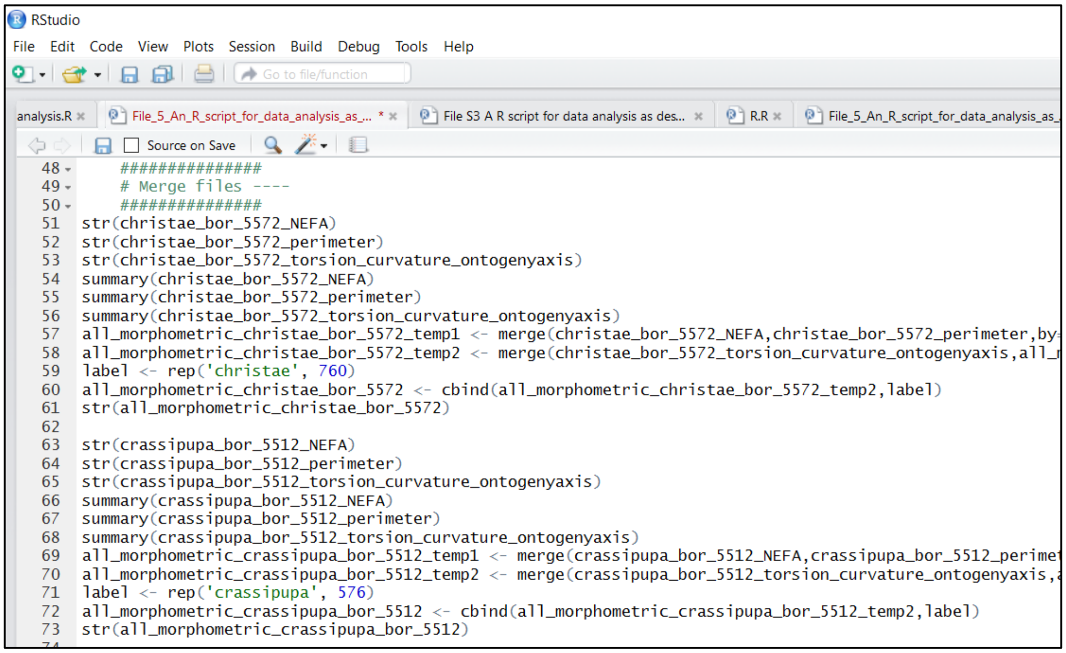

5. Define the dataset row for each shell.

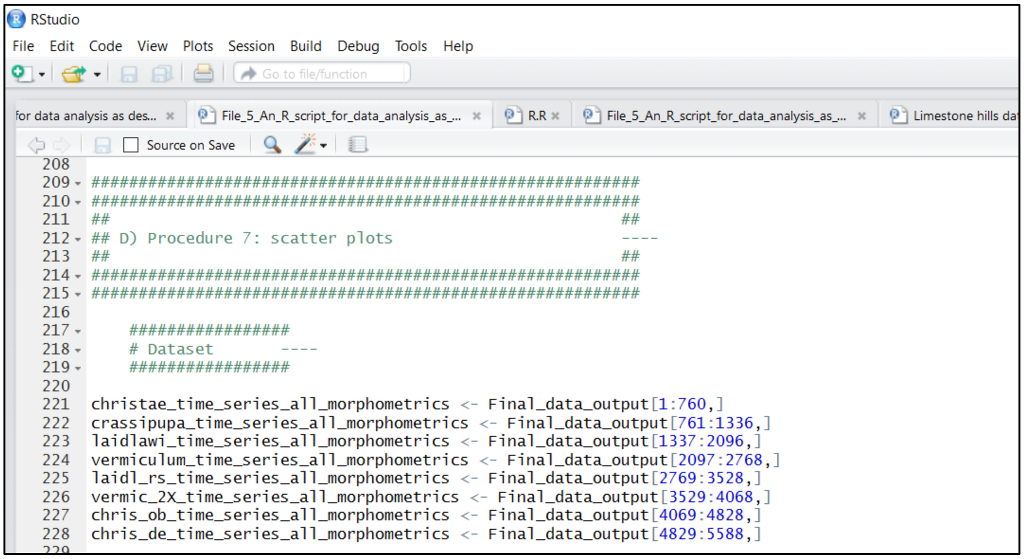

6. Select the script Parts A, B, C, and D, and execute the script (ALT+ENTER).

STEP 8: Quantitative comparison between shell forms

1. Define the dataset row for each shell.

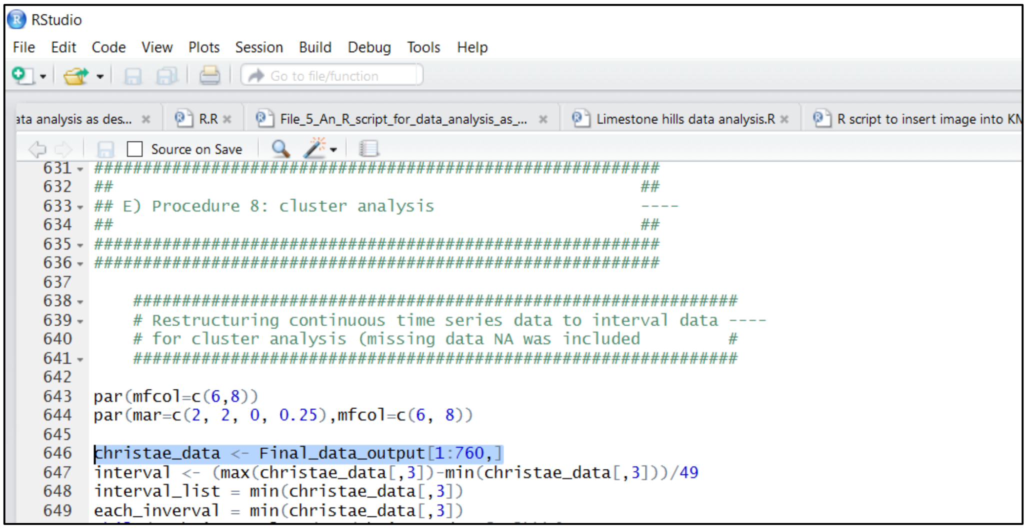

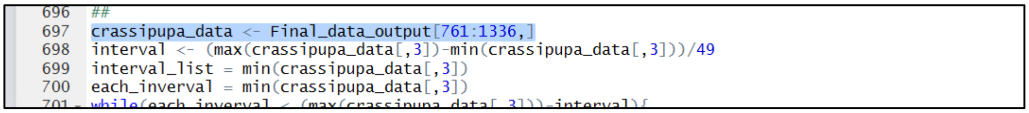

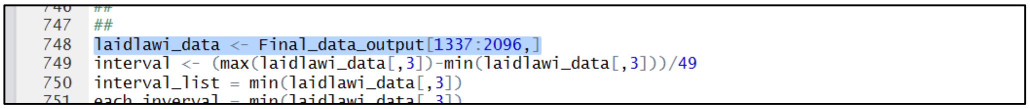

2. Define ontogeny profiles for Cluster Analysis.

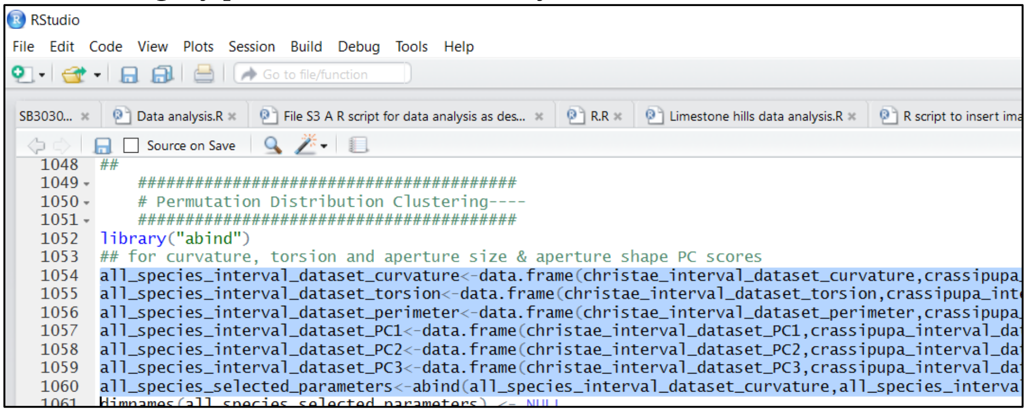

3. Label output with 3D shell name.

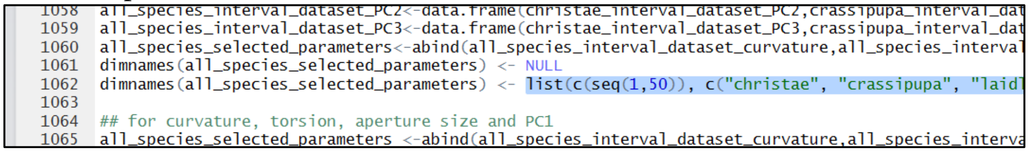

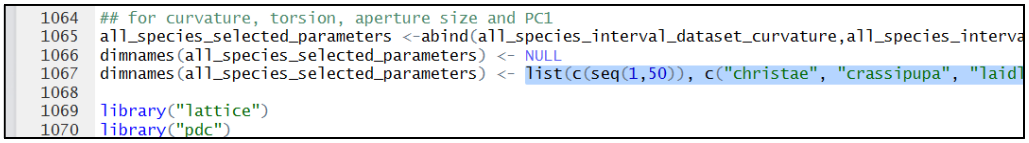

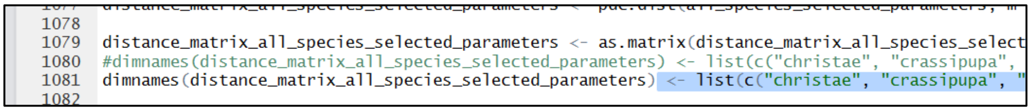

4. Select the script Part E, and execute the script (ALT+ENTER).
